# Mechanism of Action of Oxazoline‐Based Antimicrobial Polymers Against *Staphylococcus aureus*: In Vivo Antimicrobial Activity Evaluation

**DOI:** 10.1002/adhm.202301961

**Published:** 2023-09-06

**Authors:** Matilde Concilio, Ramón Garcia Maset, Laia Pasquina Lemonche, Vito Kontrimas, Ji‐Inn Song, Santhosh Kalash Rajendrakumar, Freya Harrison, C. Remzi Becer, Sébastien Perrier

**Affiliations:** ^1^ Department of Chemistry University of Warwick Coventry CV4 7AL UK; ^2^ Warwick Medical School University of Warwick Coventry CV4 7AL UK; ^3^ Department of Physics and Astronomy University of Sheffield Sheffield S3 7RH UK; ^4^ School of Life Sciences University of Warwick Coventry CV4 7AL UK; ^5^ Faculty of Pharmacy and Pharmaceutical Sciences Monash University Parkville Victoria 3052 Australia

**Keywords:** antimicrobial materials, polyoxazolines, *S. aureus*

## Abstract

Antimicrobial‐resistant pathogens have reached alarming levels, becoming one of the most pressing global health issues. Hence, new treatments are necessary for the fight against antimicrobial resistance. Synthetic nanoengineered antimicrobial polymers (SNAPs) have emerged as a promising alternative to antimicrobial peptides, overcoming some of their limitations while keeping their key features. Herein, a library of amphiphilic oxazoline‐based SNAPs using cationic ring‐opening polymerization (CROP) is designed. Amphipathic compounds with 70% cationic content exhibit the highest activity against clinically relevant *Staphylococcus aureus* isolates, maintaining good biocompatibility in vitro and in vivo. The mechanism of action of the lead compounds against *S. aureus* is assessed using various microscopy techniques, indicating cell membrane disruption, while the cell wall remains unaffected. Furthermore, a potential interaction of the compounds with bacterial DNA is shown, with possible implications on bacterial division. Finally, one of the compounds exhibits high efficacy in vivo in an insect infection model.

## Graphical abstract

Synthetic nanoengineered antimicrobial polymers (SNAPs) offer an alternative to antimicrobial peptides against multidrug‐resistant pathogens. Herein, amphiphilic oxazoline‐based SNAPs with a 70% cationic content show a potent antimicrobial activity against *S. aureus* and in vivo using an insect‐infection model. Moreover, the copolymers exhibit high biocompatibility. The compounds cause a direct membrane disruption effect against bacterial membranes and potentially target bacterial DNA.

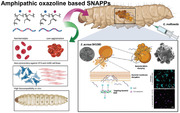

## 1 Introduction

Antimicrobial resistance (AMR) has been declared by the World Health Organization (WHO) as one of the major public health threats facing humanity.^[^ ^1^ ^]^ The emergence and spread of antibiotic‐resistant pathogens and, in particular, of the *ESKAPE* pathogens (i.e.*, Enterococcus faecium, Staphylococcus aureus, Klebsiella pneumoniae, Acinetobacter baumannii, Pseudomonas aeruginosa*, and *Enterobacter* species) have been predicted to cause more than 10 million deaths annually by 2050.^[^ ^2^ ^]^ As an alternative to common antibiotics, antimicrobial peptides (AMPs) have been extensively investigated. Briefly, AMPs are mainly defined as short cationic peptides (12–50 amino acids) with a positive net charge (+2 to +9).^[^ ^3^ ^]^ The peptide chains normally adopt an amphipathic structure due to the presence of cationic and hydrophobic moieties. This chemical structure is the key parameter inducing bacterial membrane disruption by electrostatic interaction with the negatively charged phospholipids of the bacterial membranes.^[^ ^3^ ^]^ The ability to disrupt bacterial membranes makes AMPs to have broad spectrum activity against Gram‐positive, Gram‐negative, fungi, and viruses. Recently, some AMPs have exhibited multiples mechanism of action against bacteria, binding to intracellular targets, such as DNA, RNA, and proteins, altering many bacterial cellular processes, such as metabolic pathways and cell division.^[^ ^4^, ^5^, ^6^ ^]^ Furthermore, AMPs have shown the potential to act as immuno‐modulators playing an important role during infections.^[^ ^7^ ^]^ Their several reported mechanisms of action made AMPs ideal candidates to tackle the AMR crisis, since the emergence of resistance should be less likely to occur.^[^ ^8^ ^]^

However, AMPs have displayed some limitations, such as low stability toward enzyme degradation,^[^ ^9^ ^]^ mammalian cytotoxicity especially against red blood cells,^[^ ^10^, ^11^ ^]^ and a high manufacturing cost.^[^ ^12^ ^]^ In addition, the antimicrobial activity of AMPs is massively influenced by physiological conditions.^[^ ^13^, ^14^ ^]^ For example, changes in pH, ions, cations, proteins, lipids, and serum, and the presence of red blood cells have been shown to affect the activity of AMPs, resulting in a drastic reduction in the antimicrobial performance of AMPs in vitro. Hence their success in clinical trials has been limited.^[^ ^15^ ^]^ Therefore, synthetic peptides designed in silico or by mathematical modeling have emerged as a possible alternative to natural AMPs.^[^ ^16^, ^17^ ^]^ Furthermore, synthetic nanoengineered antimicrobial polymers (SNAPs) have gained increasing interest as a promising alternative to AMPs to overcome some of their limitations while keeping their key features.^[^ ^2^, ^18^, ^19^ ^]^

Very recently, Tiller and coworkers have shown that poly(2‐oxazoline)s can be added to antimicrobial peptides to enhance the peptide activity,^[^ ^20^, ^21^, ^22^ ^]^ whilst Liu and coworkers have shown that poly(2‐oxazoline)s derivatives can act as antimicrobial agents themselves, although work to date was only focused on cationic homopolymers.^[^ ^23^, ^24^ ^]^ According to the research conducted by Liu et al., positively charged glycine‐pendant poly(2‐oxazoline) homopolymers were found to be active against *S. aureus* strains, with a mechanism of action (MOA) that targets DNA. However, when the alkyl spacer between the backbone and the cationic charge was increased, a shift in the mechanism of action from DNA targeting to membrane targeting was observed. Considering one of the key parameters in AMPs activity is their amphipathic structure, especially the hydrophobic/cationic balance, there is a definite gap in knowledge in assessing how this balance affects the MOA of poly(2‐oxazoline)s against bacteria.^[^ ^15^ ^]^ In the past decades, water‐soluble poly(2‐oxazoline)s have gained an increasing interest in the biomedical field, especially as an alternative to poly(ethylene glycol) (PEG).^[^ ^25^, ^26^ ^]^ Poly(2‐oxazoline)s are promising substitutes to PEG for biomedical applications, as the overuse of the latter has led to growing concerns in terms of toxicity, including the oxidative degradation of the main chain with the formation of toxic compounds, the stimulation of anti‐PEG IgM antibody response and, more recently, allergic reactions.^[^ ^27^ ^]^ The properties of poly(2‐oxazoline)s can be tuned easily, by variation of the side chain of the starting monomer or by the introduction of functional groups in both the chain‐ends or side‐chains. Furthermore, the use of CROP to synthesize 2‐oxazoline leads to well‐defined polymers, with narrow molecular weight distributions, high‐end‐group fidelity, and precise macromolecular architectures.

Considering the very promising potential of poly(2‐oxazoline)s as antibacterial agents, we have embarked on a systematic study assessing the structure‐property relationship underlying their antibacterial activity, for instance, the hydrophobic/cationic balance in the polymer structure, as well as defining a range of protocols to optimize the screening of material activity. We built a library of amphipathic oxazoline‐based copolymers via CROP, and evaluated their antimicrobial activity against five relevant strains of *S. aureus*, a well‐known pathogen responsible for many critical infections,^[^ ^28^, ^29^ ^]^ showing potent antimicrobial activity in an in vivo‐like wound infection medium. Their cytotoxicity was evaluated in vitro against mammalian cells and in vivo using an insect model. Two lead compounds were selected, and their mechanism of action was investigated using time‐killing experiments and several microscopy techniques, indicating that the presence of hydrophobic moieties plays a significant role in their MOA in comparison with the cationic homopolymer control. Finally, the optimized polymer compositions exhibited a potent antimicrobial activity against an in vivo insect infection model.

## 2 Results and Discussion

### 2.1 Synthesis and Characterization of 2‐Oxazoline Homopolymer and Statistical Copolymers

**Figure 1:**
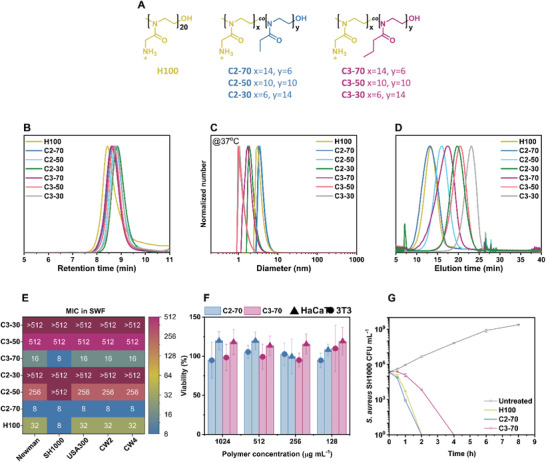
A) Chemical structure of the H100 cationic homopolymer, and of the C2‐XX and C3‐XX copolymer series. B) GPC traces of the NHBocOx homopolymer and its copolymers with EtOx or PrOx using THF as eluent. C) Normalized number DLS measurements of 1 mg mL^−1^ solutions in PBS of the deprotected polymers at 37 °C. D) HPLC chromatograms of the deprotected polymers with a gradient of 5% to 95% MeOH in 40 min using a 100 mm C18 column. E) Heatmap of the MIC values (µg mL^−1^) of the polymers tested against five different strains of *S. aureus* in SWF. F) Viability of HaCaT (triangle) and 3T3 (circle) cell lines in the presence of C2‐70 (blue) and C3‐70 (magenta) measured by the reduction of XTT. The viability was calculated from the positive control (6% DMSO  =  0% viability) and the negative control (PBS  =  100% viability). The average of three independent experiments ± standard deviation is shown. G) Time‐killing curves of S. aureus SH1000 in caMHB after exposure to H100 (yellow), C2‐70 (blue), and C3‐70 (magenta) at 2× MIC. The average of three replicates ± standard deviation is shown. SD bars are smaller than the symbols.

A library of 2‐oxazoline (co)polymers mimicking AMPs was synthesized to evaluate the effect of the cationic and hydrophobic content on their antimicrobial activity and toxicity (**Figure** **1**A). Boc‐protected amino‐2‐oxazoline (NHBocOx) was used as the cationic monomer (Figures S1 and S2, Supporting Information), and the hydrophobicity was adjusted by copolymerization with EtOx or PrOx (Figures S3 and S4, Supporting Information). The molecular weight of the (co)polymers was carefully selected to have good biocompatibility since similar antimicrobial homopolymers with higher DPs exhibited higher toxicity against cells.^[^ ^23^ ^]^ Therefore, the degree of polymerization (DP) was kept constant at 20 for all polymers, while the ratio between the cationic and the more hydrophobic monomers was varied as shown in Table S1 (Supporting Information). For clarity, the polymers are defined with “H” and “C” to represent homopolymers and copolymers, respectively, followed by the number of carbon atoms in the alkyl chain of 2‐alkyl‐2‐oxazoline (2 for ethyl, and 3 for propyl), and the number represents the percentage of cationic content in the copolymer (30, 50, 70, or 100). According to the ^1^H NMR spectra a quantitative monomer conversion was obtained for all polymerizations (Figures S5–S10, Supporting Information). CROP resulted in well‐defined polymers, as indicated by the symmetrical monomodal molecular weight distributions, with relatively high dispersity values (1.25–1.40) due to possible gel permeation chromatography (GPC) column interactions of NHBocOx repeat units (Figure 1B). This could also be observed in the GPC chromatogram of the homopolymer, which exhibited the highest polydispersity. Similar results have been previously obtained for NHBocOx homopolymers with different DPs.^[^ ^23^ ^]^ Kinetic studies of the copolymerization of NHBocOx and 2‐ethyl‐2‐oxazoline (EtOx) confirmed the synthesis of statistical copolymers with apparent reactivity ratios close to 1.00 (Figure S11 and Table S2, Supporting Information). The Boc‐protected amines were successfully deprotected via carbamate hydrolysis under acidic conditions, yielding positively charged (co)polymers (Figures S12 and S13, Supporting Information). It is important to note that due to the presence of charged moieties and their interaction with the GPC column, the (co)polymers could not be analyzed via GPC after deprotection.

Due to the amphiphilic nature of poly(2‐oxazoline) copolymers,^[^ ^30^ ^]^ we studied the solution behavior of the obtained polymers under physiological conditions via dynamic light scattering (DLS) and turbidity measurements were performed via UV–vis spectroscopy. The copolymers were dissolved at a concentration of 1 mg mL^−1^ in phosphate buffer solution (PBS, pH  =  7.4) and analyzed by DLS at 25 and 37 °C. The DLS measurements showed assemblies with diameters between 1 and 10 nm for all copolymers at both temperatures, indicating that no self‐assembly occurred under these conditions (Figure 1C and Figures S14 and S15, Supporting Information). The copolymer solutions were also analyzed by UV–vis spectroscopy, which showed that the polymers did not exhibit any thermoresponsive behavior with transmittance values close to 100% over the analyzed temperature range (Figure S16, Supporting Information).

Since it is known that both the antimicrobial activity and the toxicity towards mammalian cells are strongly influenced by the hydrophobicity of the tested compounds,^[^ ^31^, ^32^, ^33^ ^]^ the overall hydrophobicity of the polymers was also measured via HPLC.^[^ ^33^ ^]^ The polymers exhibited the expected elution trend, with the more hydrophobic copolymers eluting later compared with the more hydrophilic ones (Figure 1D). H100 and C2‐70 eluted at a similar time, indicating that the copolymerization of NHBocOx with 30% of EtOx did not affect the overall hydrophobicity of the copolymer, followed by C2‐50, C3‐70, C2‐30, C3‐50, and, lastly, the most hydrophobic C3‐30 (Table S1, Supporting Information).

### 2.2 Antimicrobial Activity Against *S. aureus*

The antimicrobial activity of the polymers was evaluated against the Gram‐positive bacterium *S. aureus*, an opportunistic member of the *ESKAPE* group associated with several acute and chronic infections including skin and soft tissue wounds.^[^ ^34^ ^]^ The gene expression, phenotypic characteristics, and metabolic state of bacteria can be influenced by the metabolites supplied (growth medium conditions), influencing the activity of the tested antimicrobial compounds.^[^ ^35^, ^36^, ^37^ ^]^ Therefore, we screened the antimicrobial activity of the polymers in standard laboratory conditions using cation‐adjusted Mueller–Hinton broth (caMHB), to compare to other studies, and in a medium mimicking the environment of a chronic wound infection, synthetic wound fluid (SWF), in order to closer mimic a wound infection microenvironment.^[^ ^38^ ^]^ Media composition affects the metabolic state of bacteria and their susceptibility toward antibiotics.^[^ ^35^, ^36^, ^37^ ^]^ Furthermore, different strains of the same specie might have different susceptibility toward antimicrobial agents.^[^ ^39^ ^]^ Therefore, we included five different strains of *S. aureus*, comprising two strains of methicillin‐sensitive (MSSA) *S. aureus* (Newman and SH1000),^[^ ^40^ ^]^ one methicillin‐resistant (MRSA) strain (USA300 LAC), and two clinical isolates from chronic wound infections from human patients (CW2 and CW4).

In the case of the copolymers in caMHB, a clear correlation between the MIC values and the cationic content was observed (Figure S17, Supporting Information). The copolymers having a higher amount of amine‐containing oxazoline monomer (C2‐70 and C3‐70) resulted in lower MIC values (32‐64 µg mL^−1^) against all the selected *S. aureus* strains compared to the more hydrophobic copolymers, which resulted in higher MICs (128–512  µg mL^−1^) or were completely inactive at concentrations tested (MIC > 512  µg mL^‐1^). These results demonstrated that the presence of a high amount of cationic monomer (70%) was necessary to achieve a good antimicrobial activity, as previously reported by Zhou et al. for purely cationic homopolymers with different DPs.^[^ ^23^ ^]^ However, the positively charged homopolymer H100 exhibited MIC values slightly higher than the two best‐performing copolymers (C2‐70 and C3‐70), confirming that hydrophobicity played a crucial role in the antimicrobial activity of the compounds. Interestingly, most of the tested polymers enhanced their antimicrobial activity in SWF, showing lower MIC values compared with caMHB (Figure 1E; Figure S17, Supporting Information), resulting in an improved antimicrobial activity for C2‐70, C3‐70 and H100 with a two‐ to four fold decrease in the MIC values. However, this trend was not observed in the case of the most hydrophobic compounds (C2‐50, C3‐50, and C3‐30). This might have been caused by the interaction of the more hydrophobic compounds with the proteins present in the medium, which hindered the improvement of their activity.^[^ ^41^, ^42^ ^]^ Interestingly, the polymers did not show a noticeable difference in antimicrobial activity depending on the *S. aureus* strain.

We hypothesized that the cationic units were necessary for the electrostatic interaction with the negatively phospholipids of the bacterial membrane and the hydrophobic moieties might promote the insertion of the polymer into the lipidic bilayer causing a membrane disruption effect. In fact, our results showed that the cationic/hydrophobic balance plays a crucial role on the antimicrobial activity of the polymeric compounds and that it needs to be assessed depending on the polymer type. Furthermore, they highlighted the importance of assessing the antimicrobial activity of polymers in media that better mimic the environment of specific infections as it can considerably vary from the results obtained in a standard testing medium.^[^ ^43^ ^]^

### 2.3 In Vitro and in Vivo Biocompatibility of the Antimicrobial of 2‐Oxazoline Polymers

Due to the amphipathic nature of the copolymers, a possible interaction with the membrane of mammalian cells could occur and cause toxicity. Therefore, to evaluate the biocompatibility of the polymers and their toxicity against mammalian cells and their possible interaction with the cell membrane, we first investigated their haemocompatibility by studying their hemolysis and haemagglutination profiles against sheep red blood cells (RBCs). In general, the polymers did not cause any significant lysis and agglutination of RBCs (Figure S18, Supporting Information). Only H100 and C2‐70 showed a mild cell agglutination at the highest concentration tested (1024  µg mL^−1^). The molecular weight of the copolymers was carefully selected to avoid low biocompatibility since similar antimicrobial homopolymers with higher DPs showed higher toxicity profiles against RBCs.^[^ ^23^ ^]^

**Figure 2:**
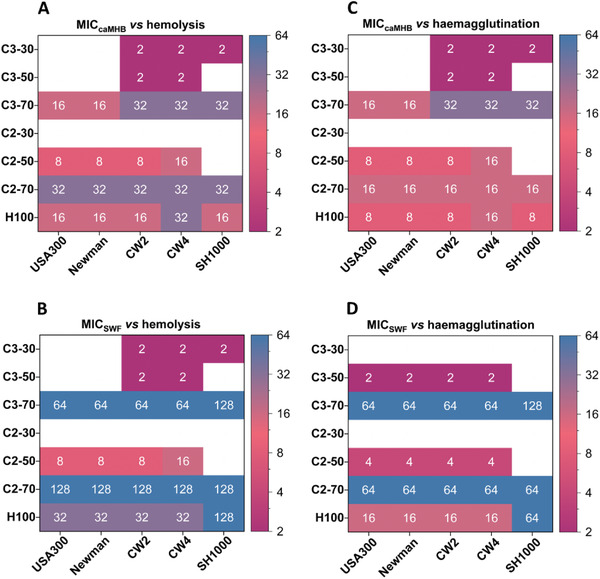
Selectivity indexes of the polymers against five *S. aureus* strains calculated from the ratio between MIC and hemolysis results in A) caMHB and B) SWF, and from the ratio between MIC and haemagglutination results in C) caMHB and D) SWF. The color gradient was used to highlight the lowest (magenta) and the highest (blue) selectivity indexes and the inactive compounds (white).

Based on our MIC results and the biocompatibility against RBCs, we calculated the therapeutic indexes (see ESI) to select the lead candidates for further investigations. By increasing the cationic content to 70%, the copolymers showed improved therapeutic indexes in comparison with the copolymers with a lower cationic content of 30% and 50% (**Figure** **2**). Also, the H100 homopolymer showed a good therapeutic index, however, the C2‐70 and C3‐70 copolymers outperformed H100 with a two‐ and four fold increase in their therapeutic indexes considering the MICs in SWF.

Further, the cytotoxicity of the two leading compounds, C2‐70 and C3‐70, was evaluated against two different mammalian cell lines. As previously mentioned, *S. aureus* is one of the most abundant pathogens on the skin associated with wound infections, leading to chronicity of the infection and wound healing impairment.^[^ ^44^ ^]^ Since keratinocytes are the major component of the epidermis, and fibroblast cells contribute to the formation of connective tissues, we selected keratinocyte (HaCaT) and fibroblast (3T3) cell lines to evaluate the cytotoxicity of the two copolymers using a metabolic mitochondrial viability (XTT) assay. The two copolymers did not cause a reduction in cell viability (Figure 1F), exhibiting a high biocompatibility toward the two cell lines even at the highest concentration tested.

### 2.4 Investigation of the Mechanism of Action of C2‐70 and C3‐70 Copolymers Against *S. aureus*

We then decided to investigate the mechanism of action (MOA) of the two lead compounds. Previously, Zhou et al. have reported that amine‐containing oxazoline homopolymers targeted DNA as their principal MOA.^[^ ^23^ ^]^ Hence, we included the H100 homopolymer in the study as a direct comparison with the copolymers. By introducing some hydrophobicity, we hypothesized that the MOA of the copolymers might rely on membrane disruption as well as potentially targeting DNA, as already reported for other antimicrobial cationic polymers.^[^ ^24^, ^33^, ^45^, ^46^, ^47^ ^]^ *S. aureus* SH1000 was selected as our model bacteria strain since the pathogen is commonly used to investigate the MOA of antibiotics against Gram‐positive bacteria.^[^ ^40^ ^]^

We first performed a time‐killing assay in caMHB to establish how fast the compounds caused bacterial killing (Figure 1G). Over 99.9% of bacteria were killed by H100 and C2‐70 by 2 h after exposure at 2× MIC, with similar killing profiles between the polymers. The more hydrophobic copolymer C3‐70 exhibited a slower killing rate (over 99.9% of *S. aureus* SH1000 killed by 4 h after exposure to 2× MIC) in comparison with C2‐70 and H100. The differences observed between the polymers might have been caused by the different hydrophobicity profiles. It might be possible that the presence of a higher hydrophobic content in C3‐70 might have potentially caused a slower insertion into the bacterial membrane, requiring a longer time to cause bacterial cell disruption compared to the more hydrophilic compounds.

**Figure 3:**
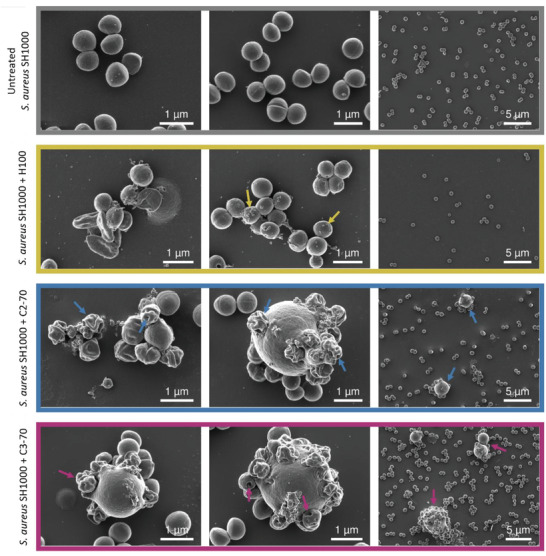
Representative scanning electron micrographs of *S. aureus* SH1000 untreated (grey) and treated with H100 (yellow), C2‐70 (blue), and C3‐70 (magenta) at 2× MIC concentration. Superficial blebs (yellow arrows), crumpled cells, membrane holes, and large spherical aggregates (blue and magenta arrows) could be observed.

Therefore, we sought to investigate the effect of the polymers on bacterial cell morphology using scanning electron microscopy (SEM). Since these experiments were performed using a higher bacterial density than in the MIC and time‐killing assays, we evaluated the effect on bacterial viability of 1 h exposure to the polymeric compounds (2× MIC) at the highest bacterial density of ≈10^8^ CFU mL^−1^ (Figure S19, Supporting Information). After 1 h treatment, H100 caused a 3‐log_10_ CFU reduction. In the case of the copolymers, the CFU did not decrease after 1 h exposure. It is important to note that the MIC of H100 in caMHB (64 µg mL^−1^) was double that of the copolymers (32 µg mL^−1^). Therefore, a higher concentration of H100 was used compared with C2‐70 and C3‐70, and this might have influenced the killing observed. From the SEM analysis, we observed a striking difference in the number of bacterial cells between the copolymers and the homopolymer (**Figure** **3**). Fewer cells were observed in the case of H100, confirming its faster bactericidal activity compared with C2‐70 and C3‐70. Since high bacteria concentrations were necessary for imaging purposes, there was a certain degree of heterogeneity in the effect of the treatments on bacteria at the concentration of polymers tested due to an inoculum effect, as previously reported for AMPs.^[^ ^48^ ^]^ Focusing on morphologically affected bacterial cells, we could observe dead cells and a roughening effect on the bacterial surface when treated with H100, with the appearance of superficial blebs (Figure 3, yellow arrows). The blebs formation has already been observed in *S. aureus* after the treatment with AMPs and SNAPs.^[^ ^47^, ^49^ ^]^ Interestingly, the cells treated with the copolymers presented strikingly different phenotypes compared with the homopolymer. “Crumpled” dead cells presenting holes on the membrane could be observed together with the appearance of enlarged spherical agglomerates (Figure 3, blue and magenta arrows). Similar phenotypes of pore formation caused by AMPs have been reported against *S. aureus*.^[^ ^50^ ^]^ The enlarged spherical aggregates were only observed after the copolymer treatment. We hypothesized that the enlarged spherical structures (≈5 times the size of untreated bacteria) could be caused by a) bacterial cell aggregates embedded in a polymeric matrix, b) debris of dead cells held together by the fixing agents of the sample preparation, or c) a single enlarged cell resulting from a possible osmotic shock due to the membrane disruption caused by the polymeric materials. However, we could not determine its nature from the SEM analyses. Nevertheless, a significant difference in the MOA between the copolymers and the homopolymer was observed, indicating that the presence of the hydrophobic moieties induced greater membrane damage compared to the purely cationic polymer. Additionally, in the case of H100, cell death already occurred after 1 h of treatment (3‐log_10_ CFU reduction) prior to the imaging and, consequently, it was difficult to determine if the membrane disruption was caused directly by the interaction of the polymeric material or as a consequence of cell death attributed to a possible different mechanism of action of the compound. In the case of the C2‐70 and C3‐70 copolymers, we could conclude that membrane disruption was directly related to the MOA of the compounds (no drastic CFU reduction).

**Figure 4:**
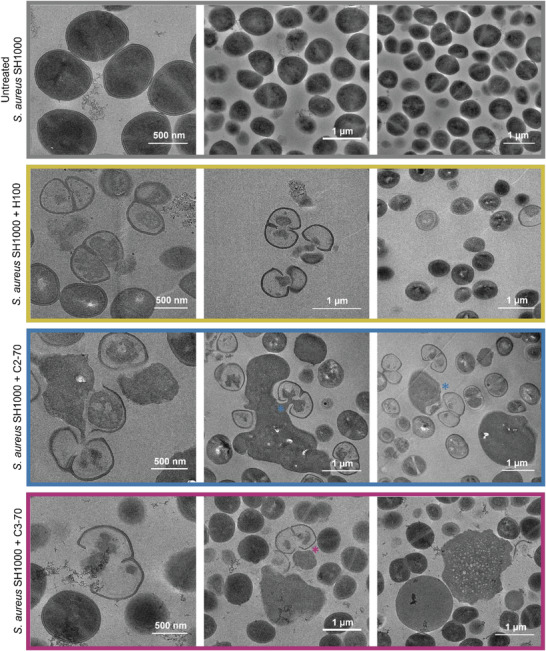
Representative transmission electron micrographs of *S. aureus* SH1000 untreated (grey) and treated with H100 (yellow), C2‐70 (blue), and C3‐70 (magenta) at 2× MIC concentration. Blue and magenta * indicate dead dividing cells and cell debris. Dark and light cells correspond to cells with and without cytoplasm, respectively.

To better understand the unexpected phenotype of *S. aureus* after the exposure to the copolymers, we performed thin‐section transmission electron microscopy (TEM) analysis of individual bacterial cells after 1 h exposure to 2× MIC of the polymeric treatments (**Figure** **4**). As can be observed, the homopolymer H100 caused cell lysis, since disrupted membranes and less electronic dense cells due to the leakage of the cytoplasmic material could be visualized. Furthermore, fewer cells and no aggregation or enlarged cells were observed in agreement with the SEM analysis. For both copolymers, cell lysis was especially observed in dividing cells. We hypothesized that the copolymers could affect the bacterial membrane, particularly in the cells that are dividing. As reported by Zhou et al., homopolymers of the cationic oxazoline directly affected DNA.^[^ ^23^ ^]^ However, Dai et al. demonstrated that homopolymers with a longer alkyl spacer between the backbone and the cationic charge switched their antibacterial mechanism from DNA‐targeting to membrane‐targeting.^[^ ^24^ ^]^

Therefore, the introduction of hydrophobicity in the copolymers might have switched their MOA, resulting in bacterial membrane disruption. However, DNA and cell division could still be two of the main targets of the copolymers since the dividing cells seemed to be more affected (Figure 4, blue and magenta). In the case of the enlarged spherical structures, the TEM analysis revealed highly electronic dense areas around lysed cells. Based on these observations, the hypothesis of single enlarged cells was discarded. On the contrary, it seemed to support the theory of cell aggregation, with the electronic dense material possibly being debris of dead cells.

**Figure 5:**
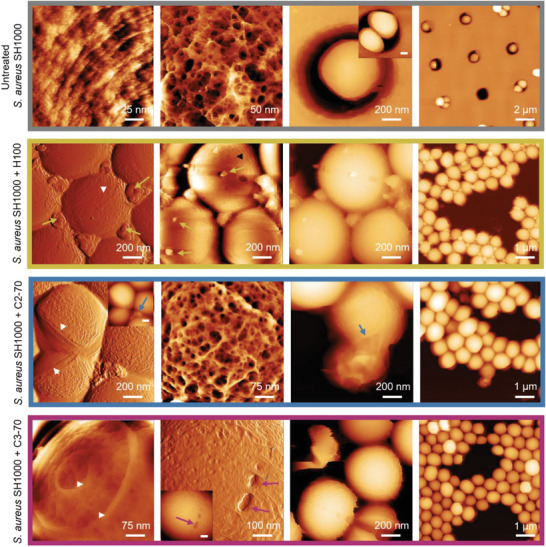
Representative AFM images of *S. aureus* SH1000 untreated (grey) and treated with H100 (yellow), C2‐70 (blue), and C3‐70 (magenta) at 2× MIC concentration. Yellow, blue, and magenta arrows indicate effects of the respective polymers on the cells, black arrowhead indicates mature peptidoglycan architecture, and white arrowhead indicates nascent peptidoglycan as a form of concentric rings.

We then sought to investigate the effect of the polymers on the bacterial cell wall nanometric morphology using atomic force microscopy (AFM). As in the EM analysis, these experiments were performed using a higher bacterial density than in the MIC assays. We prepared the sample by exposing the cells for 1 h to the polymeric compounds (2× MIC), which were then collected, boiled (to avoid using fixing agents), and attached to a flat surface for AFM imaging. From the AFM analysis, the main observation was the lack of generalized striking defects in the major component of the cell wall, the peptidoglycan (**Figure** **5**). The untreated cells showed either groups of cells or individual cells, which had the expected peptidoglycan architecture of living cells despite being boiled.^[^ ^51^ ^]^ Therefore, any effect seen in the other samples could be attributed to the polymeric treatments rather than the boiling process. Cells treated with H100 presented the same effect seen by SEM, the appearance of superficial blebs (yellow arrows). Looking at the nanostructure on the zoom images, we propose that these blebs were probably broken membranes or leaked cytoplasm that has been left out after the cells died. The peptidoglycan architecture remained mostly unchanged as the mature mesh (black arrowheads) and nascent concentric rings (white arrowheads) were still present in the majority of the cells. The C2‐70 and C3‐70 treatments did not induce the formation of “non‐peptidoglycan blebs”. As previously observed in the EM analysis, also in this case there was a certain degree of heterogeneity in the effect of the treatment on bacteria at the concentrations of polymers tested. Focusing on the morphologically affected bacteria, a major disruption was observed that could be related to exposed cytoplasm around the rupture of the cell wall (blue arrow on the C2‐70 treated cells). For both C2‐70 and C3‐70, the peptidoglycan architecture remained mostly unchanged with both mesh and rings present in the majority of the cells. The enlarged spherical agglomerates were not observed with AFM, which corroborates the hypothesis that they are aggregates of cell debris held together by fixing agents used during the SEM and TEM analyses. Given the lack of fixing during the AFM sample preparation, only the individual cells were observed on the surface. It is worth mentioning that some unusual protrusions on the nascent rings (white arrowheads on the C3‐70 images) were observed. As well as deep holes on several cells treated with C3‐70 (magenta arrows), which have been observed before on untreated cells during the division process.^[^ ^51^ ^]^ However, here this feature was more prevalent than normal, which could indicate that the cells were stuck during the division process due to the polymer activity. To conclude, the AFM analysis reinforced the hypothesis that the polymeric treatments did not cause evident damage on the cell wall, meaning they probably have a mode of action either related to lipid membrane disruption or DNA‐targeting.

**Figure 6:**
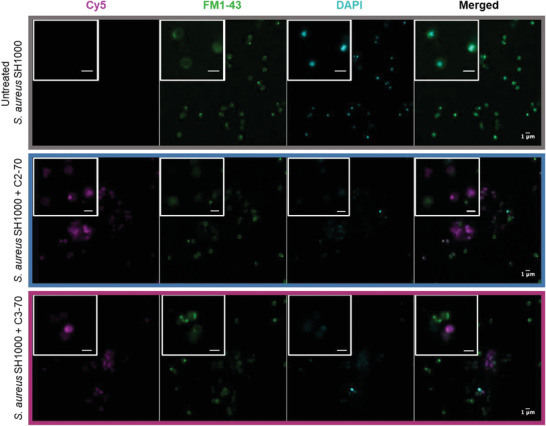
Representative confocal micrographs of S. aureus SH1000 untreated (grey), and treated with Cy5‐C2‐70 (blue) and Cy5‐C3‐70 (magenta) at 2× MIC concentration, the nucleic acid was stained with DAPI and the phospholipid bilayer was stained with FM1‐43.

Since EM analyses revealed that the copolymers caused lysis of dividing cells, we investigated the colocalization of the copolymers in *S. aureus* SH1000 using confocal microscopy. The copolymers were labeled with the Cy5‐dye by an azide‐alkyne “click” reaction (Figures S20 and S21, Supporting Information). Bacteria were incubated with the Cy5‐copolymers (magenta signal) for 1 h (2× MIC), the lipidic bilayer was stained with the dye FM1‐43 (green signal), and the bacterial DNA was stained with DAPI (cyan signal). Subsequently, the cells were fixed with 4% formaldehyde solution in PBS for 1 h prior to the imaging. As can be observed in **Figure** **6**, the untreated control showed a strong signal for the FM1‐43 and DAPI dyes, indicating healthy bacterial cells. In the case of the bacteria treated with the Cy5‐copolymers, almost all the bacterial cells showed a reduction of DAPI signal (Figure 6 and Figure S22, Supporting Information). We hypothesized that the cationic moieties of the copolymers could electrostatically interact with the negatively charged phosphate groups of the DNA, preventing the intercalation of the DAPI‐dye. Similar polymeric materials have been investigated as gene delivery agents, therefore, the binding of DNA in bacterial cells due to the amphipathic nature of the compounds is not surprising.^[^ ^52^, ^53^ ^]^

In some bacterial cells exposed to the polymeric treatment, the FM1‐43 and DAPI signals were depleted, while a strong signal from the Cy5‐copolymers was observed. This correlates with our previous observation in the AFM analysis, indicating that the compounds have a direct effect on the bacterial lipidic bilayer. These results reinforced our hypothesis that the copolymers might cause membrane disruption and affect cell division by targeting or complexing DNA. Furthermore, aggregates of bacteria could be observed similarly to the SEM and TEM analysis. The bacterial aggregates showed a strong Cy5 signal, while the signal of FM1‐43 was reduced, indicating that they contained cell debris (highly electron‐dense material observed in the TEM imaging) lacking phospholipids from the bacterial membrane. This observation reinforced our hypothesis that the spherical aggregates could be composed of bacterial cell debris.

### 2.5 In Vivo Infection Insect Model: *Galleria melonella*

Based on the promising results obtained from the XTT assays, we decided to evaluate the toxicity of the two copolymers using an in vivo invertebrate model. Invertebrate models have been used as an alternative to small mammals due to the similarities in their innate immune response,^[^ ^54^ ^]^ the easy experiment setup, the low cost associated, and the ethical benefits, resulting in reproducible and reliable outcomes comparable to those performed on mammals.^[^ ^55^ ^]^ Among all, the larvae of the greater wax moth, *Galleria mellonella*, have been widely used as an infection model and for testing the pharmacokinetics and toxicity of antimicrobial agents.^[^ ^56^, ^57^, ^58^, ^59^, ^60^, ^61^, ^62^ ^]^ Therefore, we used *G. mellonella* larvae to assess the in vivo cytotoxicity and to evaluate the in vivo antimicrobial activity of the two lead compounds. The polymers were tested at three different concentrations (20× MIC, 10× MIC, and 5× MIC) on six larvae per treatment and their effect was monitored over 7 days before the larvae entered in the pupation state where the experiment was terminated.

**Figure 7:**
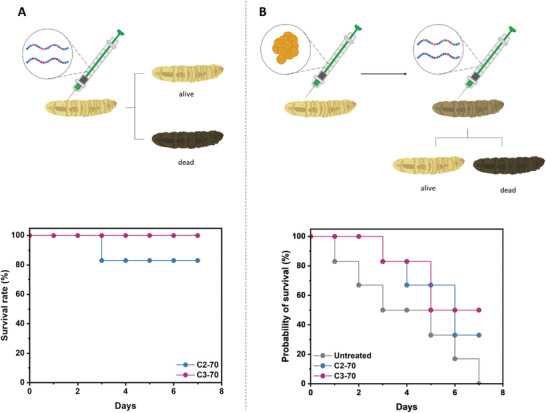
Survival rate of *G. mellonella* larvae evaluated over a period of 7 days after A) the inoculation of C2‐70 (blue) and C3‐70 (magenta) at a concentration of 20× MIC, and B) the inoculation of 108 *S. aureus* SH1000 cells followed by C2‐70 (blue) and C3‐70 (magenta) treatments at 10× MIC. Six larvae were used for each treatment.

Both C2‐70 and C3‐70 showed good biocompatibility even at the highest concentration tested (**Figure** **7**A). In the case of C2‐70, one larva died after 3 days, reducing the survival rate from 100% to 83%. However, this could also potentially be caused by the mode of injection, via a needle. A good correlation between the cytotoxicity of the compounds against mammalian cells and the invertebrate model was obtained.

Furthermore, we investigated the antimicrobial activity of the copolymers against an in vivo infection model. The larvae were infected with *S. aureus* SH1000 (10^8^ CFUs) followed by the injection of the polymeric material. The polymer dose was adjusted to 10× MIC (320 µg mL^−1^) based on the in vitro cytotoxicity where no cell death was reported. The larvae treated with only bacteria died in a constant manner over 7 days (Figure 7B). However, the addition of C2‐70 and C3‐70 increased the survival rate to 33% and 50%, respectively. The obtained results showed a promising bactericidal effect of the compounds in an in vivo infection insect model, increasing the survival rate from 0% up to 50%.

## 3 Conclusion

In conclusion, we have synthesized a library of oxazoline‐based antimicrobial polymers via CROP by varying the ratio between the cationic and hydrophobic content by polymerizing the positively charged amine‐containing oxazoline with EtOx or PrOx. The polymers did not exhibit any thermoresponsive behavior as demonstrated by DLS and turbidity measurements, and they displayed the expected trend of amphiphilicity as shown by HPLC analysis. We then tested the antimicrobial activity of the polymers via MIC assays against five strains of *S. aureus* in standard caMHB medium and in a more advanced medium mimicking the environment of a chronic wound infection (SWF). In general, the copolymers with a higher amount of cationic content resulted in lower MIC values against all the tested *S. aureus* strains compared with the more hydrophobic copolymers, demonstrating that the presence of a high amount of positively charged oxazoline was necessary to achieve a good antimicrobial activity. Furthermore, the cationic homopolymer (**H100**) exhibited slightly higher MIC values compared with the two best‐performing copolymers (i.e., **C2‐70** and **C3‐70**), confirming that hydrophobicity is a key parameter of the antimicrobial activity of the compounds. Interestingly, enhanced activity was observed for the more hydrophilic polymers in SWF, while the interaction between the hydrophobic moieties and the proteins present in the medium might have hindered the activity of the most hydrophobic compounds. Subsequently, we tested the hemocompatibility of the compounds against sheep RBCs. The polymers did not cause lysis of the cells even at the highest concentration tested, while a minor agglutination was observed for **H100** and **C2‐70** at the highest concentration.

We then selected two lead compounds based on the determined selectivity indexes and we investigated their cytotoxicity in vitro against keratinocyte (HaCaT) and fibroblast (3T3) cell lines. The compounds did not show any toxicity even at the highest concentration tested, crucial for their further application.

Finally, using several microscopy techniques, we investigated the potential membrane disruption caused by **H100**, **C2‐70**, and **C3‐70** against *S. aureus* SH1000. The amphipathic copolymers showed bacterial aggregates, membrane disruption, and binding to the bacterial DNA potentially affecting cell division. In the case of the homopolymer control, a fast‐killing effect was observed at the concentration tested, with blebs formation on the cell surface and cell lysis. In a recent publication, it has been reported that amine‐containing oxazoline copolymers target bacterial DNA.^[^ ^23^ ^]^ The incorporation of hydrophobic monomers in a statistical manner seemed to tune the mechanism of action of the compounds. For instance, membrane disruption and potential pore‐formation, as indicated in the EM imaging, were reported. Furthermore, from the confocal microscopy imaging, the penetration of the copolymers into the cytoplasm of the bacteria preventing the DAPI staining seemed to indicate that the copolymer might be binding to DNA due electrostatic interactions. Poly(oxazoline) copolymers have already been explored for gene delivery by complexing RNA or DNA,^[^ ^63^, ^64^, ^65^, ^66^ ^]^ hence the potential effect of the copolymers on cell division is not surprising. Furthermore, the AFM analysis revealed that the peptidoglycan architecture was not damaged upon the treatment with the polymers. However, membrane disruption and debris of cells caused by the amphiphilic copolymers were observed, while the homopolymer caused blebs formation, as reported in the SEM.

Finally, we investigated the cytotoxicity and the antimicrobial activity of the lead compounds in vivo using the larvae *Galleria mellonella* as an insect model. The compounds exhibited high biocompatibility even at the highest concentration tested and the most amphipathic copolymer (C3‐70) showed a good antimicrobial activity in vivo against *S. aureus* SH1000 infection in the larvae, increasing their viability from 0% up to 50%. These results are promising for the evaluation of their toxicity and antimicrobial activity in more advanced in vivo testing.

In summary, we have shown that amphipathic poly(2‐oxazoline)‐based SNAPs had a potent antimicrobial activity against clinical isolates of *S. aureus*, and the mechanism of action of the materials was based on bacterial membrane disruption and potentially targeted bacterial nucleic acids as well, without compromising the bacterial cell wall. Furthermore, the compounds showed promising antimicrobial activity in vivo using an insect model. In the future, the application of the poly(2‐oxazoline) SNAPs as topical agents to prevent wound infections and prosthetic joint infections will be investigated. Furthermore, the investigation at the molecular level of SNAPs‘ interactions with lipidic bilayer might lead to a better understating of their MOA and to the improvement of the next generation of materials to aid the fight against the AMR crisis.

## Conflict of Interest

The authors declare no conflict of interest.

## Author Contributions

M.C. and R.G.M. contributed equally to this work, conceived the study, performed most of the experiments, and composed the manuscript. L.P.L. performed atomic force microscopy imaging. V.K. acquired the confocal microscopy images. J.‐I.S. performed the cytotoxicity experiments in HaCaT and 3T3 cell lines. S.K.R. edited the manuscript and aided in the *G. mellonella* experiments. S.P., F.H., and C.R.B. contributed essential resources, oversaw all aspects of the study execution, and edited the manuscript. All authors contributed to the generation, analysis, or interpretation of the data and edited the manuscript.

## Supporting information

Supporting Information

## Data Availability

The data that support the findings of this study are available from the corresponding author upon reasonable request.

## References

[1] Antimicrobial resistance, https://www.who.int/news‐room/fact‐sheets/detail/antimicrobial‐resistance (accessed: September 2022).

[2] N. F. Kamaruzzaman , L. P. Tan , R. H. Hamdan , S. S. Choong , W. K. Wong , A. J. Gibson , A. Chivu , M. F. Pina , Int. J. Mol. Sci. 2019, 20, 2747. 31167476 10.3390/ijms20112747 PMC6600223

[3] N. Mookherjee , M. A. Anderson , H. P. Haagsman , D. J. Davidson , Nat. Rev. Drug Discovery 2020, 19, 311. 32107480 10.1038/s41573-019-0058-8

[4] R. E. W. Hancock , M. A. Alford , E. F. Haney , Nat. Rev. Microbiol. 2021, 19, 786. 34183822 10.1038/s41579-021-00585-w

[5] J. Li , J. J. Koh , S. Liu , R. Lakshminarayanan , C. S. Verma , R. W. Beuerman , Front Neurosci 2017, 11, 73. 28261050 10.3389/fnins.2017.00073 PMC5306396

[6] M. H. Cardoso , B. T. Meneguetti , B. O. Costa , D. F. Buccini , K. G. N. Oshiro , S. L. E. Preza , C. M. E. Carvalho , L. Migliolo , O. L. Franco , Int. J. Mol. Sci. 2019, 20, 4877. 31581426 10.3390/ijms20194877 PMC6801614

[7] M. A. Alford , B. Baquir , F. L. Santana , E. F. Haney , R. E. W. Hancock , Front Microbiol 2020, 11, 1902. 32982998 10.3389/fmicb.2020.01902 PMC7481365

[8] R. Spohn , L. Daruka , V. Lázár , A. Martins , F. Vidovics , G. Grézal , O. Méhi , B. Kintses , M. Számel , P. K. Jangir , Nat. Commun. 2019, 10, 1. 31586049 10.1038/s41467-019-12364-6 PMC6778101

[9] L. Tang , J. Chen , Z. Zhou , P. Yu , Z. Yang , G. Zhong , Microbes Infect 2015, 17, 402. 25752416 10.1016/j.micinf.2015.02.005

[10] M. Bacalum , M. Radu , Int. J. Pept. Res. Ther. 2015, 21, 47.

[11] G. Laverty , B. Gilmore , SOJ Microbiol Infect Dis 2014, 2, 1.29756026

[12] B. L. Bray , Nat. Rev. Drug Discovery 2003, 2, 587. 12815383 10.1038/nrd1133

[13] L. Otvos Jr , J. D. Wade , Front Chem 2014, 2, 62. 25152873 10.3389/fchem.2014.00062 PMC4126357

[14] D. A. Holdbrook , S. Singh , Y. K. Choong , J. Petrlova , M. Malmsten , P. J. Bond , N. K. Verma , A. Schmidtchen , R. Saravanan , Biochim. Biophys. Acta – Biomembr. 1860, 2374 (2018). 10.1016/j.bbamem.2018.06.002 29885294

[15] R. E. W. Hancock , H. G. Sahl , Nat. Biotechnol. 2006, 24, 1551. 17160061 10.1038/nbt1267

[16] Y. Han , M. Zhang , R. Lai , Z. Zhang , Peptides 2021, 146, 170666. 34600037 10.1016/j.peptides.2021.170666

[17] C. Wang , S. Garlick , M. Zloh , Biomolecules 2021, 11, 471. 33810011 10.3390/biom11030471 PMC8004669

[18] A. Jain , L. S. Duvvuri , S. Farah , N. Beyth , A. J. Domb , W. Khan , Adv. Healthcare Mater. 2014, 3, 1969. 10.1002/adhm.201400418 25408272

[19] H. Etayash , R. E. W. Hancock , Pharmaceutics 2021, 13, 1820. 34834239 10.3390/pharmaceutics13111820 PMC8621177

[20] M. Schmidt , S. Harmuth , E. R. Barth , E. Wurm , R. Fobbe , A. Sickmann , C. Krumm , J. C. Tiller , Bioconjugate Chem. 2015, 26, 1950. 10.1021/acs.bioconjchem.5b00393 26284608

[21] M. Schmidt , L. K. Bast , F. Lanfer , L. Richter , E. Hennes , R. Seymen , C. Krumm , J. C. Tiller , Bioconjugate Chem. 2017, 28, 2440. 10.1021/acs.bioconjchem.7b00424 28817271

[22] J. R. Park , A. D. Verderosa , M. Totsika , R. Hoogenboom , T. R. T. Dargaville , Biomacromolecules 2021, 22, 5185. 34726387 10.1021/acs.biomac.1c01133

[23] M. Zhou , Y. Qian , J. Xie , W. Zhang , W. Jiang , X. Xiao , S. Chen , C. Dai , Z. Cong , Z. Ji , N. Shao , L. Liu , Y. Wu , R. Liu , Angew. Chem., Int. Ed. 2020, 59, 6412. 10.1002/anie.202000505 32083767

[24] C. Dai , M. Zhou , W. Jiang , X. Xiao , J. Zou , Y. Qian , Z. Cong , Z. Ji , L. Liu , J. Xie , Z. Qiao , R. Liu , J. Mater. Sci. Technol. 2020, 59, 220.

[25] A. Mero , G. Pasut , L. Dalla Via , M. W. M. Fijten , U. S. Schubert , R. Hoogenboom , F. M. Veronese , J. Controlled Release 2008, 125, 87. 10.1016/j.jconrel.2007.10.010 18031860

[26] O. Sedlacek , B. D. Monnery , S. K. Filippov , R. Hoogenboom , M. Hruby , Macromol. Rapid Commun. 2012, 33, 1648. 23034926 10.1002/marc.201200453

[27] O. Sedlacek , R. Hoogenboom , Adv. Ther. 2020, 3, 1900168.

[28] H. F. Chambers , F. R. DeLeo , Nat. Rev. Microbiol. 2009, 7, 629. 19680247 10.1038/nrmicro2200 PMC2871281

[29] B. Krismer , C. Weidenmaier , A. Zipperer , A. Peschel , Nat. Rev. Microbiol. 2017, 15, 675. 29021598 10.1038/nrmicro.2017.104

[30] R. Hoogenboom , H. Schlaad , Polym. Chem. 2017, 8, 24.

[31] K. Kuroda , W. F. DeGrado , J. Am. Chem. Soc. 2005, 127, 4128. 15783168 10.1021/ja044205+

[32] S. Liu , R. J. Ono , H. Wu , J. Y. Teo , Z. C. Liang , K. Xu , M. Zhang , G. Zhong , J. P. K. Tan , M. Ng , Biomaterials 2017, 127, 36. 28279920 10.1016/j.biomaterials.2017.02.027

[33] A. Kuroki , P. Sangwan , Y. Qu , R. Peltier , C. Sanchez‐Cano , J. Moat , C. G. Dowson , E. G. L. Williams , K. E. S. Locock , M. Hartlieb , S. Perrier , ACS Appl. Mater. Interfaces 2017, 9, 40117. 29068226 10.1021/acsami.7b14996

[34] B. P. Conlon , BioEssays 2014, 36, 991. 25100240 10.1002/bies.201400080

[35] L. M. Thoma , B. R. Boles , K. Kuroda , Biomacromolecules 2014, 15, 2933. 25010735 10.1021/bm500557d PMC4130249

[36] I. A. Ratiu , T. Ligor , V. Bocos‐Bintintan , H. Al‐Suod , T. Kowalkowski , K. Rafińska , B. Buszewski , J Breath Res 2017, 11, 036012. 28649963 10.1088/1752-7163/aa7ba2

[37] M. R. Gonzalez , V. Ducret , S. Leoni , B. Fleuchot , P. Jafari , W. Raffoul , L. A. Applegate , Y. A. Que , K. Perron , Front Cell Infect Microbiol 2018, 8, 39. 29535973 10.3389/fcimb.2018.00039 PMC5835353

[38] M. Werthen , L. Henriksson , P. Ø. Jensen , C. Sternberg , M. Givskov , T. Bjarnsholt , APMIS 2010, 118, 156. 20132180 10.1111/j.1600-0463.2009.02580.x

[39] A. R. Brochado , A. Telzerow , J. Bobonis , M. Banzhaf , A. Mateus , J. Selkrig , E. Huth , S. Bassler , J. Zamarreño Beas , M. Zietek , Nature 2018, 559, 259. 29973719 10.1038/s41586-018-0278-9 PMC6219701

[40] B. Salamaga , L. Kong , L. Pasquina‐Lemonche , L. Lafage , von und zur M. Muhlen , J. F. Gibson , D. Grybchuk , A. K. Tooke , V. Panchal , E. J. Culp , Demonstration of the role of cell wall homeostasis in Staphylococcus aureus growth and the action of bactericidal antibiotics, PNAS, 2021, Vol.118, p. e2106022118. 10.1073/pnas.2106022118 PMC8612353 34716264

[41] J. Svenson , B. O. Brandsdal , W. Stensen , J. S. Svendsen , J. Med. Chem. 2007, 50, 3334. 17569519 10.1021/jm0703542

[42] Z. Oesterreicher , S. Eberl , A. Nussbaumer‐Proell , T. Peilensteiner , M. Zeitlinger , Clin. Microbiol. Infect. 2019, 25, 759. 10.1016/j.cmi.2018.09.004 30267931

[43] D. K. Mercer , M. D. T. Torres , S. S. Duay , E. Lovie , L. Simpson , M. von Köckritz‐Blickwede , C. De la Fuente‐Nunez , D. A. O'Neil , A. M. Angeles‐Boza , Front. Cell Infect. Microbiol. 2020, 326. 32733816 10.3389/fcimb.2020.00326 PMC7358464

[44] R. D. Wolcott , J. D. Hanson , E. J. Rees , L. D. Koenig , C. D. Phillips , R. A. Wolcott , S. B. Cox , J. S. White , Wound Repair Regener. 2016, 24, 163. 10.1111/wrr.12370 26463872

[45] E. F. Palermo , I. Sovadinova , K. Kuroda , Biomacromolecules 2009, 10, 3098. 19803480 10.1021/bm900784x

[46] C. Peng , A. Vishwakarma , S. Mankoci , H. A. Barton , A. Joy , Biomacromolecules 2019, 20, 1675. 30844254 10.1021/acs.biomac.9b00029

[47] R. Garcia Maset , A. Hapeshi , S. Hall , R. M. Dalgliesh , F. Harrison , F. Harrison , S. Perrier , ACS Appl. Mater. Interfaces 2022, 22, 1429. 10.1021/acsami.2c05979 PMC9335526 35819416

[48] M. R. Loffredo , F. Savini , S. Bobone , B. Casciaro , H. Franzyk , M. L. Mangoni , L. Stella , Proc. Natl. Acad. Sci. U.S.A. 2021, 118, e2014364118. 34021080 10.1073/pnas.2014364118 PMC8166072

[49] X. Wu , Z. Wang , X. Li , Y. Fan , G. He , Y. Wan , C. Yu , J. Tang , M. Li , X. Zhang , Antimicrob. Agents Chemother. 2014, 58, 5342. 24982064 10.1128/AAC.02823-14 PMC4135812

[50] F. Armas , S. Pacor , E. Ferrari , F. Guida , T. A. Pertinhez , A. A. Romani , M. Scocchi , M. Benincasa , PLoS One 2019, 14, e0212447. 30789942 10.1371/journal.pone.0212447 PMC6383929

[51] L. Pasquina‐Lemonche , J. Burns , R. D. Turner , S. Kumar , R. Tank , N. Mullin , J. S. Wilson , B. Chakrabarti , P. A. Bullough , S. J. Foster , Nature 2020, 582, 294. 32523118 10.1038/s41586-020-2236-6 PMC7308169

[52] M. N. Leiske , F. H. Sobotta , F. Richter , S. Hoeppener , J. C. Brendel , A. Traeger , U. S. Schubert , Biomacromolecules 2017, 19, 748. 10.1021/acs.biomac.7b01535 29261298

[53] P. Gurnani , A. K. Blakney , R. Terracciano , J. E. Petch , A. J. Blok , C. R. Bouton , P. F. McKay , R. J. Shattock , C. Alexander , Biomacromolecules 2020, 21, 3242. 32644777 10.1021/acs.biomac.0c00698

[54] K. Kavanagh , E. P. Reeves , FEMS Microbiol. Rev. 2004, 28, 101. 14975532 10.1016/j.femsre.2003.09.002

[55] L. Guilhermino , T. Diamantino , M. C. Silva , A. M. V. M. Soares , Ecotoxicol. Environ. Saf. 2000, 46, 357. 10903834 10.1006/eesa.2000.1916

[56] B. B. Fuchs , E. O'Brien , J. B. El Khoury , E. Mylonakis , Virulence 2010, 1, 475. 21178491 10.4161/viru.1.6.12985

[57] C. J.‐Y. Tsai , J. M. S. Loh , T. Proft , Virulence 2016, 7, 214. 26730990 10.1080/21505594.2015.1135289 PMC4871635

[58] D. Romera , J. J. Aguilera‐Correa , M. García‐Coca , I. Mahillo‐Fernández , L. Viñuela‐Sandoval , J. García‐Rodríguez , J. Esteban , Pathog Dis 2020, 78, ftaa067. 33098293 10.1093/femspd/ftaa067

[59] R. J. Thomas , K. A. Hamblin , S. J. Armstrong , C. M. Müller , M. Bokori‐Brown , S. Goldman , H. S. Atkins , R. W. Titball , Int. J. Antimicrob. Agents 2013, 41, 330. 23402703 10.1016/j.ijantimicag.2012.12.009

[60] R. Maguire , O. Duggan , K. Kavanagh , Cell Biol. Toxicol. 2016, 32, 209. 27122324 10.1007/s10565-016-9329-x

[61] K. Ignasiak , A. Maxwell , BMC Res. Notes 2017, 10, 1. 28851426 10.1186/s13104-017-2757-8 PMC5576310

[62] K. Kavanagh , G. Sheehan , J. Fungi 2018, 4, 113. 10.3390/jof4030113 PMC6162640 30235800

[63] G. H. Hsiue , H. Z. Chiang , C. H. Wang , T. M. Juang , Bioconjugate Chem. 2006, 17, 781. 10.1021/bc050317u 16704218

[64] M. Hartlieb , D. Pretzel , K. Kempe , C. Fritzsche , R. M. Paulus , M. Gottschaldt , U. S. Schubert , Soft Matter 2013, 9, 4693.

[65] E. Vlassi , S. Pispas , Macromol. Chem. Phys. 2015, 216, 873.

[66] E. Haladjova , M. Smolíček , I. Ugrinova , D. Momekova , P. Shestakova , Z. Kroneková , J. Kronek , S. Rangelov , J. Appl. Polym. Sci. 2020, 137, 49400.

