# Supplementary material for: Mechanism of Action of Oxazoline‐Based Antimicrobial Polymers Against Staphylococcus aureus: In Vivo Antimicrobial Activity Evaluation
Source: Adv Healthc Mater. 2023 Sep 6;12(29):2301961. doi: 10.1002/adhm.202301961 (PMC11468764; doi:10.1002/adhm.202301961)
Supplement: Supplementary file 1 — Supporting Information [file ADHM-12-2301961-s001.pdf]

ADVANCED
HEALTHCARE
MATERIALS

Supporting Information

for *Adv. Healthcare Mater.*, DOI 10.1002/adhm.202301961

Mechanism of Action of Oxazoline-Based Antimicrobial Polymers Against *Staphylococcus aureus*: In Vivo Antimicrobial Activity Evaluation

Matilde Concilio, Ramón Garcia Maset, Laia Pasquina Lemonche, Vito Kontrimas, Ji-Inn Song, Santhosh Kalash Rajendrakumar, Freya Harrison, C. Remzi Becer and Sébastien Perrier**

ELECTRONIC SUPPORTING INFORMATION

Mechanism of action of oxazoline-based antimicrobial polymers against *S. aureus: in vivo* antimicrobial activity evaluation

Matilde Concilio[†a], *Ramón Garcia Maset*[†b], *Laia Pasquina Lemonche*[c], *Vito Kontrimas*[b], *Ji-Inn Song*[b], *Santhosh Kalash Rajendrakumar*[b], *Freya Harrison*[d], *C. Remzi Becer**[b], *Sébastien Perrier**[a,b,e]

[a] Warwick Medical School, University of Warwick, Coventry, CV4 7AL, UK

[b] Department of Chemistry, University of Warwick, Coventry, CV4 7AL, UK

[c] Department of Physics and Astronomy, University of Sheffield, Sheffield, S3 7RH, UK

[d] School of Life Sciences, University of Warwick, Coventry, CV4 7AL, UK

[e] Faculty of Pharmacy and Pharmaceutical Sciences, Monash University, Parkville, Victoria 3052, Australia

Email: *S.Perrier@warwick.ac.uk and remzi.becer@warwick.ac.uk*

Materials

2-Ethyl-2-oxazoline (99 +%, Acros Organics, EtOx) was dried over calcium hydride and distilled under reduced pressure prior to use. Methyl tosylate (98%, Aldrich, MeTos) and propargyl tosylate (97%, Acros Organics, PrTos) were distilled under reduced pressure, stored over molecular sieves and under nitrogen. Butyronitrile (≥ 99%), zinc acetate dihydrate (≥ 98%, $Zn(OAc)_2$ 2 H_2O), triethylamine (≥ 99%), 4-dimethylaminopyridine (≥ 99%, DMAP), copper (I) bromide (98%, Cu(I)Br), methanol (≥ 99.8%, MeOH), methanol for HPLC (≥ 99.9%), diethyl ether (≥ 99.5%), dichloromethane (≥ 99%, DCM), Dulbecco's modified Eagle's medium (DMEM), Müller-Hinton Broth type II (MHB cationic adjusted, caMHB), phosphate buffered saline (PBS) tablets, Triton X, buffered peptone water, resazurin sodium salt, concanavalin A from Canavalia ensiformis (Jack bean), and 12-well plate Corning® Costar® TC-treated multiple well plates were purchased from Sigma-Aldrich. Ethanolamine (≥ 99%), extra dry *N*,*N*-dimethylacetamide (99.5%, DMAc), extra dry acetonitrile (99.9 +%, CH_3CN), magnesium sulfate ($MgSO_4$), potassium hydroxide (KOH), sodium chloride (≥ 99.5%, NaCl), tetrahydrofuran (≥ 99.5%, THF), Corning™ Costar™ flat bottom cell culture plates (flat bottom, clear, with Lid, polystyrene, 96 wells, sterile, tissue-culture treated surface), Thermo Scientific™ 96 well round (U) bottom plate, 4′,6-diamidino-2-phenylindole (DAPI), Gibco foetal bovine serum, hexamethyldisilazane (Electronic grade, 99 +%, HDMS), fixable analog of FM™ 1-43 membrane stain and poly-D-lysine were purchased from Fisher Scientific. Boc-glycine (99 +%) and 1,1,4,7,7-pentamethyldiethylenetriamine (98 +%, PMDETA) were purchased from Acros Organics. 2-Chloroethylamine hydrochloride (98 +%) and trifluoroacetic acid (> 99.5%, TFA) were purchased from Alfa Aesar. (*N*-(3-dimethylaminopropyl)-*N*'-ethylcarbodiimide) (≥ 96.0%, EDAC) and defibrinated sheep blood and round coverslip round of 12 mm (631-1577P) were purchased from VWR International Ltd (UK). Cy5-azide was purchased from Lumiprobe GMHB. Pre-wetted RC tubings MWCO 1 kD were purchased from Spectrum Labs. XTT cell proliferation assay kit (30-1011K™) was purchased from ATCC. Screw cap test tubes (polypropylene, flat bottom) were purchased from Labdirect. 29G hypodermic needles were purchased from Becton Dickinson Medical. µ-Slide 8 Well Grid-500 were purchased from Thistle Scientific. µ-Slide 8 Well Grid-500 chambers were purchased from Ibidi. Glutaraldehyde solution 25% for electron microscopy was purchased from PanReac AppliChem. *Galleria mellonela* were purchased from Livefoods, UK. The bacterial isolates used were *S. aureus* SH1000, *S. aureus* USA300 Los Angeles County clone, *S. aureus* Newman, and two *S. aureus* isolates from a chronic post-surgical wound (denoted CW2 and CW4, with thanks to Tim Sloan and Nottingham University Hospitals). The cells lines used were embryonic fibroblast (Mus musculus) 3T3 (ATCC® CRL-1658TM)) and primary human keratinocytes HaCaT (ATCC® PCS-200-011).

Synthesis of 2-propyl-2-oxazoline (PrOx)

The monomer was synthesized as previously reported in literature.[1] Butyronitrile (40 mL, 459.56 mmol), 2-amino ethanol (41.61 mL, 689.34 mmol), and $Zn(OAc)_2$ 2 H_2O (2.0175 g, 9.19 mmol) were transferred into a round bottom flask and heated to reflux at 130 °C overnight. Subsequently, the yellow-orange reaction mixture was cooled to room temperature and DCM (100 mL) was added to the flask. The organic layer was washed with distilled water (100 mL x 2) and brine (100 mL x 2), and dried over $MgSO_4$. After filtration, the solvent was evaporated under reduced pressure, and the resulting yellow oil was purified by distillation under vacuum at 50-60 °C, yielding a colourless liquid (15.41 g, 29.6% yield). ^1H NMR (400 MHz, $CDCl_3$), δ (ppm): 0.92 (3H, t, CH_3, J = 7.2 Hz), 1.61 (2H, m, $CH_2CH_2CH_3$, J = 7.5 Hz), 2.20 (2H, t, $CH_2CH_2CH_3$, J = 7.5 Hz), 3.77 (2H, t, CH_2COCH_2, J = 9.6 Hz), 4.16 (2H, t, CH_2CNCH_2, J = 9.4 Hz).

Synthesis of NHBocOx monomer

Step 1: Synthesis of *tert*-butyl (2-((2-chloroethyl)amino)-2-oxoethyl)carbamate (Intermediate 1)

N-Boc-glycine (15 g, 85.63 mmol) and 2-chloroethylamine hydrochloride (10.9250 g, 94.19 mmol,) were transferred into a round bottom flask immersed in an ice bath and dissolved in DCM (290 mL) under nitrogen atmosphere. Subsequently, TEA (24 mL, 171.25 mmol) was added to the reaction mixture, which was left stirring for 20 min. DMAP (1.0461 g, 8.56 mmol) was dissolved in DCM (10 mL) and injected into the solution, which was left under nitrogen for 30 min. Finally, EDAC (16.52 mL, 94.19 mmol) was added to the reaction mixture, which was stirred at room temperature under nitrogen overnight. The DCM was partially removed by rotary evaporation and the organic phase was washed with 0.5 M HCl aqueous solution (200 mL x3), distilled water (200 mL x3), and brine (200 mL x3). The solvent was removed under reduced pressure and the resulting product (15 g, 74% yield) was used without further purification for the second reaction step.^1H NMR (400 MHz, $CDCl_3$), δ (ppm): 1.46 (9H, s, Boc group), 3.60-3.65 (4H, m, CH_2CH_2Cl), 3.82 (2H, d, $NHCH_2CO$, , J = 5.7 Hz), 5.10 (1H, s, $OCONHCH_2$), 6.55 ppm (1H, s, CH_2CONH).

Step 2: Synthesis of *tert*-butyl ((4,5-dihydrooxazol-2-yl)methyl)carbamate (NHBocOx)

Intermediate 1 (15 g, 63.37 mmol) and KOH (7.11 g, 126.74 mmol) were dissolved in methanol (250 mL). The reaction mixture was heated to 50 °C overnight under nitrogen. Subsequently, the precipitate was filtered out and washed with DCM. The solvents were removed *via* rotary evaporator. The resulting product was redissolved in DCM and washed with distilled water (x2). The DCM was evaporated under reduced pressure and the product was dried under vacuum. The product was further purified by distillation under vacuum at 100-120 °C and stored at - 18 °C under nitrogen (9 g,

71% yield). ^1H NMR (400 MHz, $CDCl_3$), δ (ppm): 1.45 (9H, s, Boc group), 3.85 (2H, t, CH_2COCH_2, J = 9.5 Hz), 3.96 (2H, m, $NHCH_2C$), 4.31 (2H, t, CH_2CNCH_2, J = 9.5 Hz), 5.09 (1H, s, CH_2NHCO).

Synthesis of NHBocOx homopolymer *via* CROP

NHBocOx (0.5273 g, 2.63 mmol), dry DMAc (0.66 mL) and MeTos ((19.9 µL, 0.132 mmol) were transferred into a microwave vial. The reaction mixture was purged with a nitrogen flow for 30 min and reacted at 100 °C for 1.5 h.

Synthesis of NHBocOx- 2-alkyl-2-Ox random copolymers *via* CROP

All copolymers were synthesized under similar conditions. As an example, a general procedure for the synthesis of the 50:50 NHBocOx : EtOx copolymer is described. NHBocOx (0.3802 g, 1.90 mmol) and dry DMAc (0.32 mL) were transferred into a microwave vial and the reaction mixture was purged with a nitrogen flow for 30 min before adding EtOx (191.7 µL, 1.90 mmol) and dry CH_3CN (0.63 mL). The final [monomers]:[I] ratio was 20:1, and the [DMAc]:[CH_3CN] ratio was 2:1. Subsequently, MeTos (28.7 µL, 0.19 mmol) was added into the reaction mixture, which was reacted at 100 °C for

Deprotection of NHBocOx homopolymer and NHBocOx-2-alkyl-2-Ox random copolymers

The NHBocOx homopolymer and all the copolymers were deprotected under similar conditions. As an example, the deprotection of the 50:50 NHBocOx : EtOx copolymer is described. The copolymer (0.57 g) was dissolved in DCM (3 mL) in a round bottom flask. TFA (3 mL) was added in the flask and the reaction mixture was reacted at 46 °C for 3 h. The deprotected copolymer was purified by precipitation in cold diethyl ether (x 3). To replace the TFA counterion, the polymer was dissolved in water and dialyzed against NaCl solution, followed by dialysis against distilled water for 4 days. The Boc group removal and the presence of traces of the TFA counterion were monitored by ^1H and ^{19}F NMR. Finally, the dialyzed product was freeze-dried and stored at 4 °C.

Azide-alkyne "click" reaction for the dye-polymer conjugation (Cy5-C2-70 and Cy5-C3-70)

For the azide-alkyne "click" reaction with the Cy5-azide dye, the polymerization of NHBocOx (70% content) and EtOx or PrOx was initiated with PrTos to yield copolymers bearing an alkyne group at one chain-end. The polymerization of the copolymers and their deprotection were carried on as previously described. After deprotection, the copolymers were purified by precipitation in cold diethyl ether (x 3). Both deprotected copolymers were conjugated with the dye under similar conditions. As an example, a general procedure for the synthesis of the Cy5-C2-70 copolymer is described. Deprotected PrTos-initiated C2-70 (88.6 mg, 0.041 mmol) and Cy5-azide (24.8 mg, 0.041 mmol) were transferred into a microwave vial and dissolved in distilled water (0.70 mL) and THF (1.26 mL). The reaction mixture was purged with a nitrogen flow for 10 min. In the meantime, Cu(I)Br (9.9 mg) was

weighted in a second microwave vial, which was sealed and purged with nitrogen for 5 min before adding THF (825 µL). The reaction mixture was further purged for 5 min and, subsequently, PMDETA (14.44 µL) was added. After 10 min under nitrogen flow, the Cu(I)Br/PMDETA stock solution (0.14 mL) was transferred in the vial containing the polymer and the dye. The vial was immersed in an oil bath at 50 °C and reacted overnight. To replace the TFA counterion and remove the dye excess, the polymer was dissolved in water and dialyzed against NaCl aqueous solution, followed by dialysis against distilled water for 4 days.

Characterization

Nuclear magnetic resonance (NMR) spectroscopy

^{1}H, ^{13}C and ^{19}F NMR spectra were recorded on a Bruker Avance III HD 400 MHz. $CDCl_3$ and D_2O were used as solvent and the signal of the residual deuterated solvent was used as reference for the chemical shift, δ. The data analysis was performed using TopSpin 4.0.7 software.

Size exclusion chromatography (SEC)

The measurements were performed using THF (2% TEA) as eluent. The Agilent Technologies 1260 Infinity instrument was equipped with a refractive index (RI) and 308 nm UV detectors, a PLgel 5 µm guard column, and a PLgel 5 µm mixed D column (300 × 7.5 mm). Samples were run at a flow rate of 1 mL min^{-1} at 40 °C. Poly(methyl methacrylate) standards (Agilent PMMA calibration kits, M-M-10 and M-L-10) were used for the calibration. Before injection (100 µL), the samples were filtered through a PTFE membrane with 0.2 µL pore size. The data were determined by conventional calibration using Agilent GPC/SEC software.

Dynamic light scattering (DLS)

The size distributions were determined using a Litesizer 500 (Anton Paar) equipped with a 40 mW semiconductor laser diode (658 nm). Samples were prepared in PBS at 1 mg mL^{-1} and filtered through a Nylon membrane (0.2 µm pore size) before transferring to Suprasil® quartz cuvettes (Hellman, 100-QS, light path = 10.00 mm). Measurements were performed at 25 °C or 37 °C in a backscattering geometry with a detection angle of 175°. Measurements were repeated three times with an automatic attenuation selection and measurement position. The results were analyzed using Anton Paar Kalliope software.

Turbidity measurements

Turbidity analyses were performed using an Agilent Technologies Cary 3500 UV-Vis spectrophotometer equipped with an Agilent Cary UV-Vis multicell Peltier system. The measurements were carried on using Suprasil® quartz cuvettes (Hellman, 100-QS, light path = 10.00 mm) filled with

1 mg mL^{-1} polymer solutions in PBS. Two heating/cooling cycles between 20 and 50 °C were performed with a temperature gradient of 1 °C min^{-1} at λ = 600 nm. All data were recorded using the Agilent Cary UV Workstation 1.2.328 software and elaborated using Origin 2021b software.

High-performance liquid chromatography (HPLC)

HPLC chromatograms were measured using a Shimadzu Prominence HPLC equipped with a DGU-20A3 Prominence degasser, two LC-20AD Prominence liquid chromatograph, a SIL-20A Prominence autosampler, an SPD-M20A Prominence diode array detector, a CTO-20AC Prominence column oven, and a CBM-20A Prominence communications bus module. The HPLC was fitted with an Agilent Eclipse Plus C18 column (4.6 × 100 mm) with 3.5 µm packing (95 Å). Water and methanol containing 0.04 vol% TFA were used as mobile phase A and B, respectively. The gradient used for HPLC analysis was increased from 5% to 95% B in 20 min. UV detection was monitored at λ = 210 nm. Samples were dissolved in mobile phase A at 1 mg mL^{-1}.The injection volume was 50 µL. Methods were edited and run using Shimadzu LC solution online software and data analysed using Shimadzu LC solution offline software. All data were elaborated using Origin 2021b software.

Minimum inhibitory concentrations (MICs)

MICs were determined according to the standard Clinical Laboratory Standards Institute broth microdilution method.[2] Briefly, a single colony of bacteria grown on LB agar plates was picked and resuspended in cationic adjusted Mueller-Hinton broth (caMHB). The bacterial cell concentration was adjusted to a concentration of ~ 10^8 colony-forming units per mL (CFU mL^{-1}) by measuring the optical density at 600 nm (OD_{600}) to obtain 0.5 McFarland standard (OD_{600}~0.08-0.1). A further 100-fold dilution was performed to reach a bacterial concentration of 10^6 CFU mL^{-1}. Polymers were dissolved in caMHB and serial dilutions were performed in 96-well plates to yield concentrations ranging from 1024 µg mL^{-1} to 8 µg mL^{-1}, in a total volume of 50 µL. 50 µL of bacterial suspension was then added, resulting in a final bacterial density of 5 x 10^5 CFU mL^{-1}. The micro-well plates were incubated at 37 °C for 18 h. The bacterial viability was evaluated by adding 10 µL of resazurin dye in each well (final concentration of 0.5 mg mL^{-1}). The plates were incubated for 30 min at 37 °C. A noticeable change of colour could be observed for live bacterial cells (pink colour) versus dead cells (blue colour). The protocol was carried on as previously described by M. Elshikh *et al.*[3] Three independent experiments (three different days) were performed with three technical replicates per experiment. Additionally, MIC experiments were performed following the same methodology, but using synthetic wound fluid in place of caMHB (SWF; peptone water:foetal bovine serum 50:50%v/v, as described by M. Werthén *et al.*).[4]

Hemolysis assay

Sheep red blood cells (RBCs) were washed with PBS *via* centrifugation (4500 × g for 1 min) until the supernatant was clear. The polymers were dissolved in PBS and serially diluted (from 1024 mg mL^{-1} to 16 mg mL^{-1}), with a final volume of 100 µL in each well. A solution of 2%v/v Triton X-100 was used as positive control and PBS as negative control. 100 µL of 6%v/v of RBCs in PBS were added to each well of a Corning Costar TC-treated 96-well plate. The plates were incubated at 37 °C for 2 h. The 96-well plates were centrifuged at 600 × *g* for 10 min. 100 µL of the supernatant was transferred to a new plate. The absorbance at 540 nm was measured using a Cytation 3 microplate reader (Bio Tek). Three independent experiments (three different days) were performed with three technical replicates per experiment.

Haemagglutination assay

Haemagglutination assays were performed according to the protocol established by Banerjee *et al*.[5] The RBCs were prepared as described in the hemolysis assay. 50 µL of the 6%v/v RBCs solution in PBS were transferred to a each well of a Corning Costar 96-well clear round bottom TC-treated microplate. The polymers were dissolved in PBS and serially diluted from 1024 µg mL^{-1} to 16 µg mL^{-1}. 50 µL of the polymer solutions were transferred to the wells containing the RBCs. A 50 mg mL^{-1} Concanavalin A solution was used as positive control, and PBS as negative control. The microplate was incubated at 37 °C for 1 h and the haemagglutination effect was visually assessed. Three independent experiments (three different days) were performed with three technical replicates per experiment.

Determination of the therapeutic index

The therapeutic index was calculated as follow:

$$\text{Therapeutic index} = \frac{\text{Toxicity}}{\text{Antimicrobial activity}}$$

The "toxicity" corresponds to the polymer concentrations that caused hemolysis or haemagglutination, and the "antimicrobial activity" corresponds to the MIC values measured in caMHB or SWF against each S. *aureus* strains. For each polymer in a specific *S. aureus* strain, 4 therapeutic indexes could be calculated.

In vitro toxicity of antimicrobial polymers in eukaryotic cells

Cells were seeded into Cellstar TC-Treated 96-Well Plates at a density of 3 × 10^3 cells and 2 × 10^3 cells per well for 3T3 cells and HaCaT cells, respectively, and cultured at 37 °C in basal medium DMEM with 10% bovine calf serum (for 3T3 cells) or 10% foetal bovine serum (for HaCaT cells) supplemented with

penicillin/streptomycin. The cells were allowed to grow for 24 h. The medium was replaced with fresh medium and complemented with solutions of polymer (ranging from 32 to 1024 µg mL^{-1}). Fresh medium and 6% DMSO were used as negative and positive controls, respectively. Cells were further incubated for 24 h. The medium containing polymer solution was discarded, and the cells were washed 3 times with PBS. Fresh medium containing 2,3-bis-(2-methoxy-4-nitro-5-sulfophenyl)-2H-tetrazolium-5-carboxanilide) (0.2 mg mL^{-1}) and *N*-methyl dibenzopyrazine methyl sulphate (5 µM) was added and incubated for 17 h. Cells were transferred to a plate reader and absorbance at 450 and 650 nm was measured. The viability was expressed relatively to the positive (100% viability) and the negative (0% viability) controls.

Bacterial time killing experiments

From an overnight culture of *S. aureus* SH1000 in caMHB, a fresh inoculum was prepared and incubated at 37°C with shaking until a mid-exponential phase was obtained (10^{7-8} CFU mL^{-1}). The *S. aureus* SH1000 suspension was diluted to reach a bacterial concentration of 10^5-10^6 CFU mL^{-1}.The bacterial suspension was placed into 5 mL screw-cap test tubes and incubated in the presence of polymeric compounds at 2 × MIC in a final volume of 2 mL at 37 °C with shaking. *S. aureus* SH1000 was counted by taking 50 µL aliquots at regular time intervals (t = 0, 30, 120, 240, 360 and 480 min), making serial dilutions, and plating on LB agar.

For assessing killing at high bacterial densities, bacteria were grown in caMHB until OD_{600} reached 0.5 (corresponding to ~ 10^8 CFU mL^{-1}). 2 mL of the bacteria solution were aliquoted in 5 mL screw tubes and the lead compounds were added at a final concentration of 2 x MIC (final volume of 4 mL). The bacteria were incubated in the presence of the compounds for 1 h at 37 °C with shaking. The cultures were serially diluted in PBS and plated on LB agar for CFU counting.

Scanning electron microscopy

From an overnight of *S. aureus* SH1000 in caMHB, a fresh inoculum was prepared and incubated at 37 °C with shaking until mid-exponential phase was obtained (10^{7-8} CFU mL^{-1} in caMHB). This bacterial solution was incubated in the presence of polymeric compounds (2 × MIC) at 37 °C for 1 h. The cells were pelleted by centrifugation at 6000 × g for 2 min, followed by 3 washes with PBS. 12 mm diameter circular glass cover slips were incubated with 50 µL of poly-lysine in a 24-well tissue culture plate for 15 min. The poly-lysine solution was removed, and the cover slips were left to dry. The bacterial cell pellets were resuspended in 400 µL of PBS and 100 µL of this suspension were added to the cover slips. After 30 min of incubation, the excess volume was removed. The cells were fixed at 4 °C overnight with a 2.5% glutaraldehyde solution in PBS, followed by 3 washes with PBS. Dehydration

was performed using an ethanol gradient (20%, 50%, 70%, 90%, 100%, and 100% ethanol) for 10 min at each concentration. After complete dehydration, the cover slips were incubated with 0.5 mL of HDMS for 30 min. The cover slips were moved to clean wells and left to dry for 30 min. Copper tape was added to the SEM sample holders and the cover slips were placed on top. The samples were sputtered using a carbon coater (Emitech K950X). Imaging was performed at the Warwick Electron microscopy Research Technology Platform on a Zeiss Gemini Scanning Electron Microscope equipped with an InLens detector at a voltage of 1 kV.

Transmission electron microscopy

S. aureus SH1000 was grown and treated with the polymeric material (2 x MIC) in caMHB as described in the SEM section. The bacterial cells were fixed at 4 °C overnight with a 2.5% glutaraldehyde solution in PBS, followed by 3 washes with PBS. The pellets were subsequently incubated with 1% osmium tetroxide for 60 min at room temperature. Following washing with PBS, the samples were dehydrated in a graded acetone series and transferred to graded acetone:epoxy resins mixtures for 45 min at each composition until pure resin was reached, and then incubated overnight at a constant temperature. The specimens were sectioned with an ultramicrotome. Imaging was performed at the Warwick Advanced Bioimaging Research Technology Platform on a Jeol 2100Plus LaB6 transmission electron microscope equipped with a Gatan OneView IS camera.

Atomic force microscopy

From an overnight of *S. aureus* SH1000 in caMHB, a fresh inoculum was prepared and incubated at 37 °C with shaking until mid-exponential phase was obtained. This bacterial solution was incubated in the presence of polymeric compounds (2 × MIC) at 37 °C for 1 h, except for the control without any polymer. The cells were pelleted by centrifugation at 4000 × g for 5 min, followed by 3 washes with PBS. The cell suspension in PBS was transferred to 1.5 mL plastic tubes and boiled at 100 °C in a dry heat-block for 20 min. The cells were pelleted again and resuspended in 100 µL of PBS. Parallelly, 11 mm diameter circular mica discs were coated with Cell-Tak according to manufacturer' instructions. The excess of Cell-Tak was removed by washing with water and the mica discs were dried with a nitrogen flow. 50 µL of cell suspension were incubated in the coated mica for 1 h, washed with water and gently dried with a nitrogen flow. The sample was immediately imaged in liquid (10 mM Tris + 150 mM KCl) using a Bruker Dimensions Fast Scan. A Bruker Fast Scan-D AFM probe was used with a PeakForce Tapping imaging mode using no more than 2 nN of threshold force. All images were performed on the same day to avoid artefacts or differences due to different AFM probes. The images were post processed and analyzed with the open source software Gwyddion.[6]

Confocal microscopy

From an overnight of *S. aureus* SH1000 in caMHB, a fresh inoculum was prepared and incubated at 37 °C with shaking until mid-exponential phase was obtained. The bacterial solution was incubated in the presence of Cy5-conjugated polymers (2 × MIC) at 37 °C for 1 h. The cells were pelleted by centrifugation at 6000 × g for 2 min, followed by 3 washes with PBS. The bacterial solutions were stained with FM1-43X (1 µg mL^{-1}) for 15 min and DAPI for 5 min (1 µg mL^{-1}), followed by 3 washes with PBS. Other samples were just stained with DAPI for 5 min (1 µg mL^{-1}), followed by 3 washes with PBS. Chambered slides were incubated with 100 µL of poly-lysine for 15 min. The poly-lysine solution was removed, and the chambered coverslips were left to dry. The bacterial cell pellets were resuspended in 400 µL of PBS and 100 µL of this suspension were added to the chamber slides. After 1 h of incubation, the excess volume was removed. The cells were fixed at 4 °C for 1 h with 4% formaldehyde solution in PBS, followed by 3 washes with PBS. Imaging was performed on a Zeiss LSM 880 fluorescence microscope. The 100 X oil objective was used. All data were elaborated using Omero 5.6.

In vivo toxicity using an insect model (*Galleria mellonella*)

The larvae of the *Galleria mellonella* were used to assess the toxicity of polymeric compounds *in vivo* as previously reported.[7] The 5th instar larvae were maintained in sawdust at 4-8 °C for 1-3 days. Larvae were weighed and selected for the experiment (250 mg ± 10 mg). 6 larvae were used for each dose of polymeric compound tested. Larvae were placed on ice for immobilization during injections. Before the injection, the surface of the larvae was sterilized using a 70% ethanol aqueous solution. 10 µL of the polymeric solution (ranging from 320 to 640 µg mL^{-1}) were injected in the larvae proleg using a 29G hypodermic needle. PBS was used as a negative control. The larvae were placed in petri dishes in the dark at room temperature and survival was monitored for 7 days. The larvae were considered dead by visual inspection when a reduction or total impairment of mobility and a change in color to black/brown were observed.

Galleria mellonella infection

The infection assay was performed in a modified version of the protocol described by Sheehan *et al*.[8] The 5th instar larvae were maintained in sawdust at 4-8 °C for 1-3 days. Larvae were weighed and selected for the experiment (270 mg ± 10 mg). From an overnight of *S. aureus* SH1000 in caMHB, a fresh inoculum was prepared and incubated at 37 °C with shaking until mid-exponential phase was obtained. The cells were pelleted by centrifugation at 6000 × g for 2 min, followed by 3 washes with PBS. The bacterial solution was diluted to reach a bacterial concentration of 10^6 CFU mL^{-1} (OD_{600} = 0.01). Larvae were inoculated with 20 µL of the *S. aureus* SH1000 solution by injection in the last left proleg into the hemocoel using a 29G hypodermic needle. After 12 h, the polymeric solution

(10 x MIC in PBS) and untreated control (PBS) were injected into the larvae. A total of 6 larvae were used for each condition from 3 different bacterial overnights. The survival was monitored over a period of 7 days.

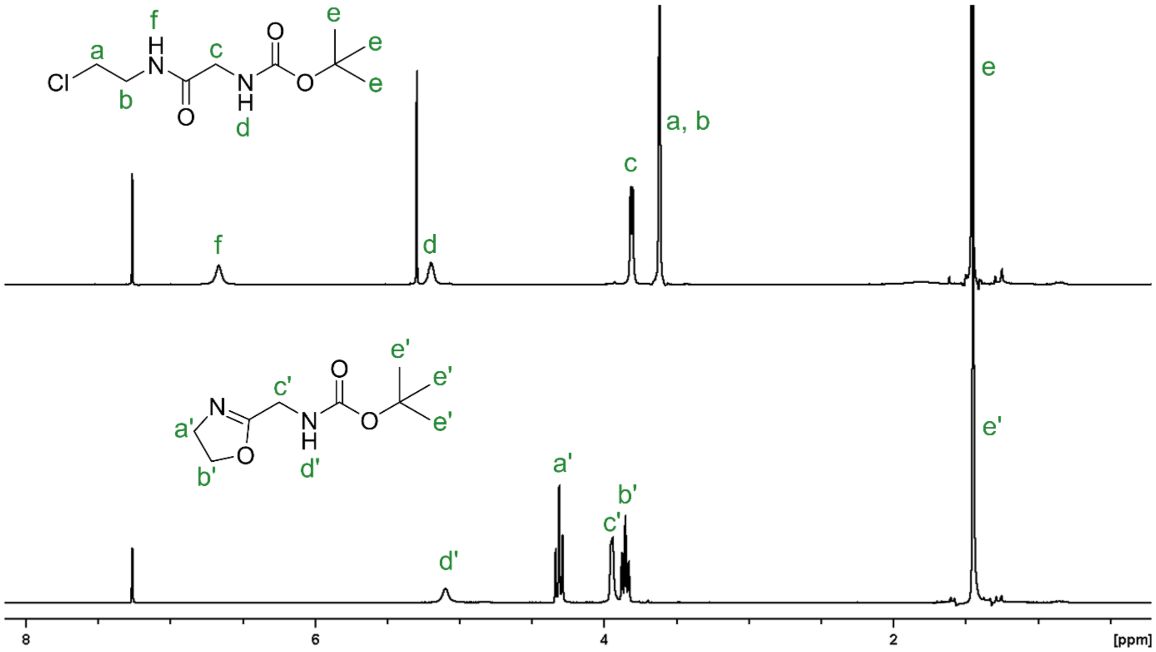

Figure S1. ^{1}H NMR spectra (400 MHz, $CDCl_3$) of the intermediate *tert*-butyl (2-((2-chloroethyl)amino)-2-oxoethyl) carbamate (top), and the purified NHBocOx monomer (bottom) .

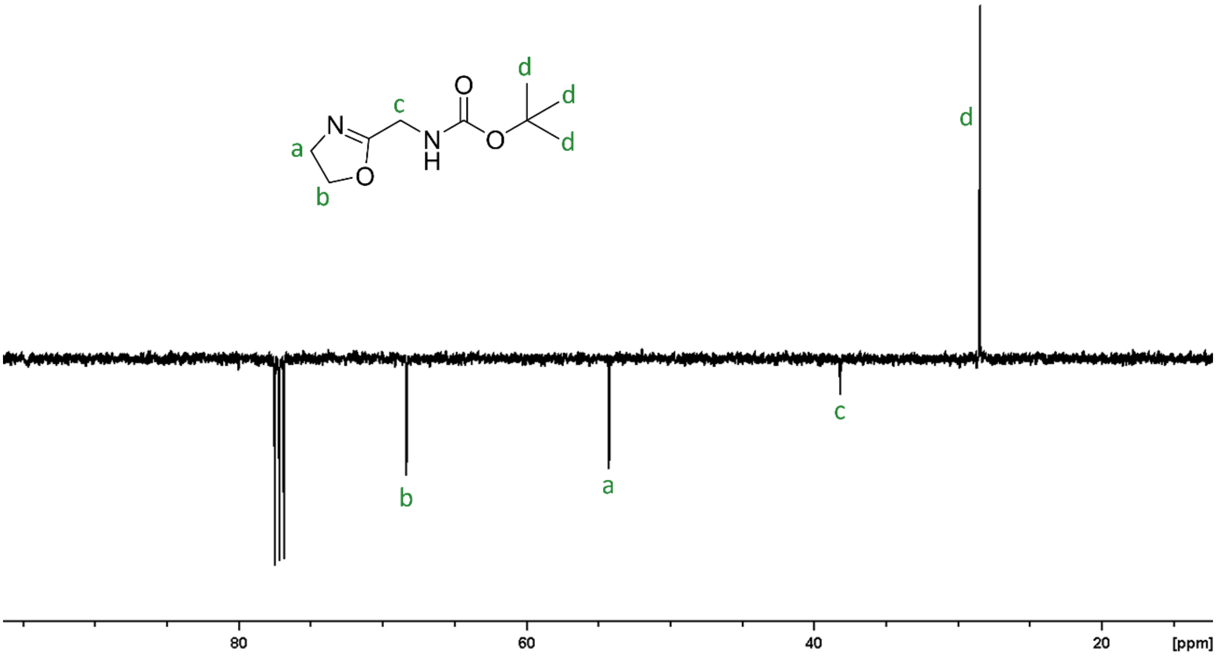

Figure S2. APT ^{13}C NMR spectrum (400 MHz, $CDCl_3$) of the Boc-protected oxazoline monomer NHBocOx.

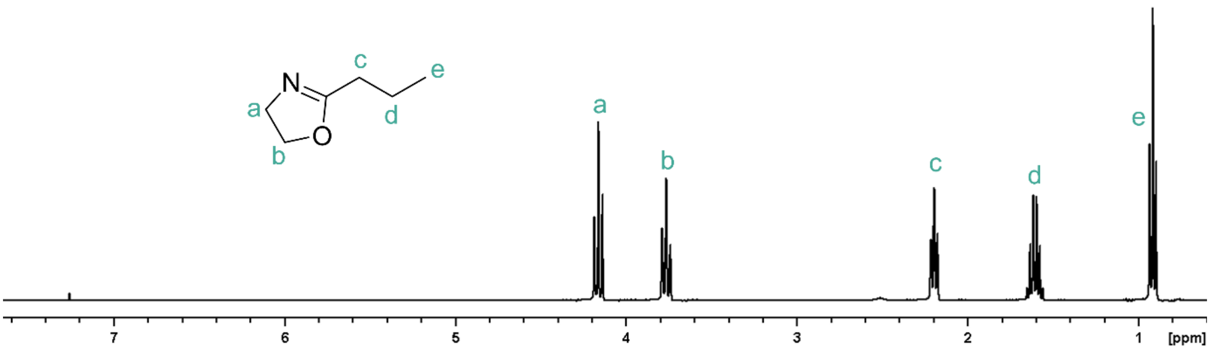

Figure S3. ^{1}H NMR spectrum (400 MHz, $CDCl_3$) of the purified PrOx monomer.

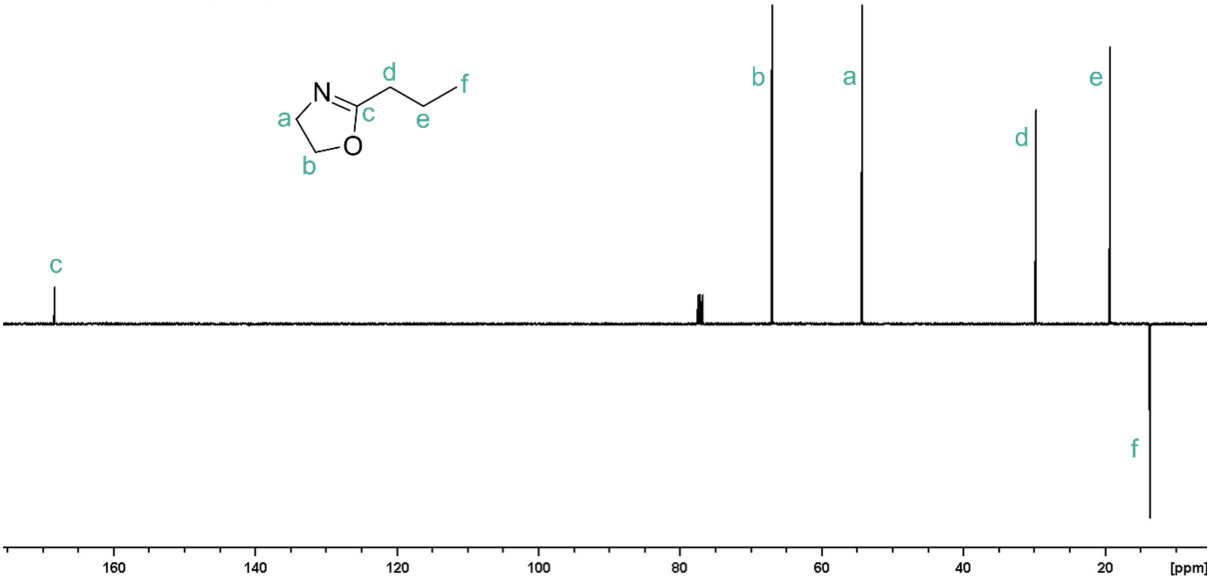

Figure S4. APT ^{13}C NMR spectrum (400 MHz, $CDCl_3$) of PrOx monomer.

Table S1. NHBocOx homopolymer and its copolymers with EtOx or PrOx obtained *via* CROP using MeTos (I) as initiator.

Sample	Hydrophobic Ox	Cationic content (%)	Ox:NHBocOx:I [a]	$M_{n,th}$ (g mol^{-1})	$M_{n,SEC}$ (g mol^{-1}) [b]	$Đ$ [b]	Elution time HPLC (min)
**H-100**	-	100	0:22:1	4420	2757	1.70	13.2
**C2-70**	EtOx	70	6:15:1	3613	3461	1.30	13.2
**C2-50**	EtOx	50	10:10:1	3009	2645	1.42	16
**C2-30**	EtOx	30	13:7:1	2690	2363	1.25	19.7
**C3-70**	PrOx	70	6:15:1	3698	3700	1.28	17.4
**C3-50**	PrOx	50	9:11:1	3236	3099	1.33	20.5
**C3-30**	PrOx	30	13:6:1	2493	2688	1.35	23.1

[a] Determined by 1H NMR, [b] determined by GPC using THF as eluent against PMMA standards.

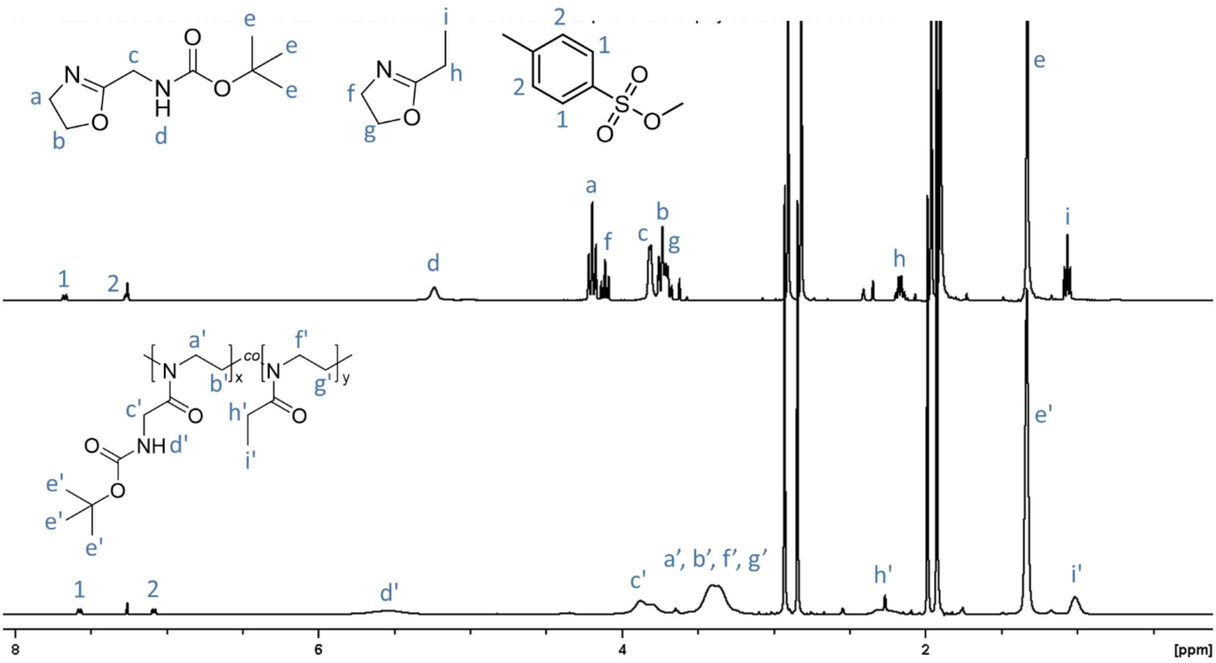

Figure S5. ^{1}H NMR spectra (400 MHz, $CDCl_3$) of C2-70 at t_0 (top) and t_f (bottom).

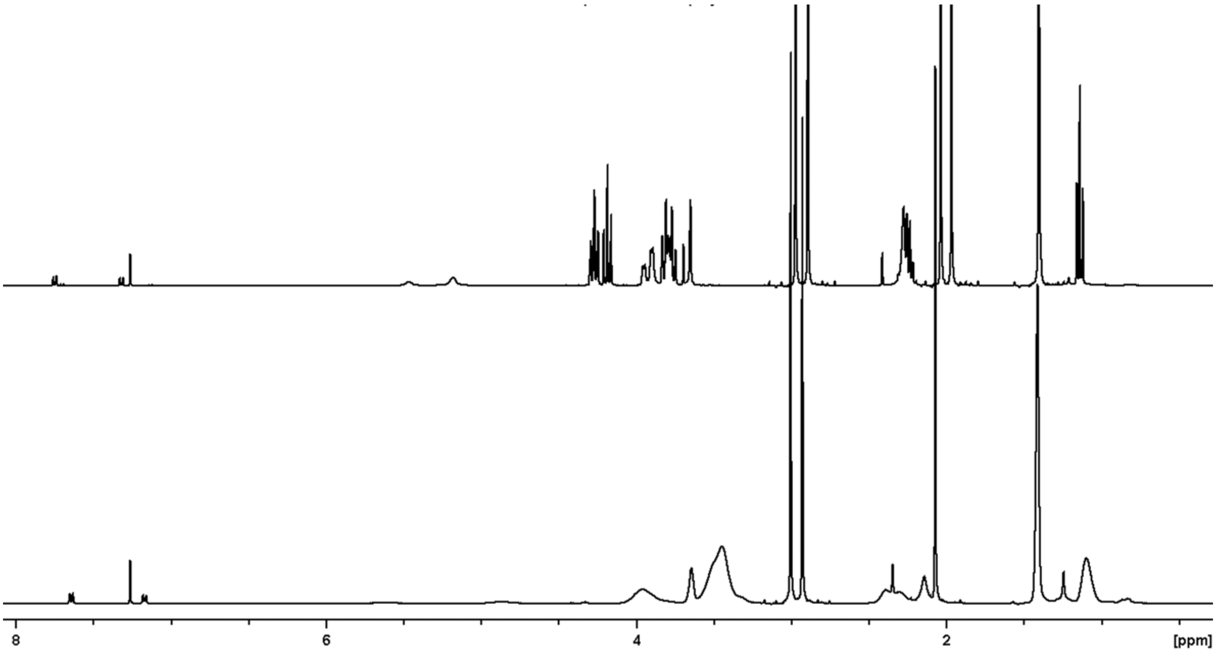

Figure S6. ^{1}H NMR spectra (400 MHz, $CDCl_3$) of C2-50 at t_0 (top) and t_f (bottom).

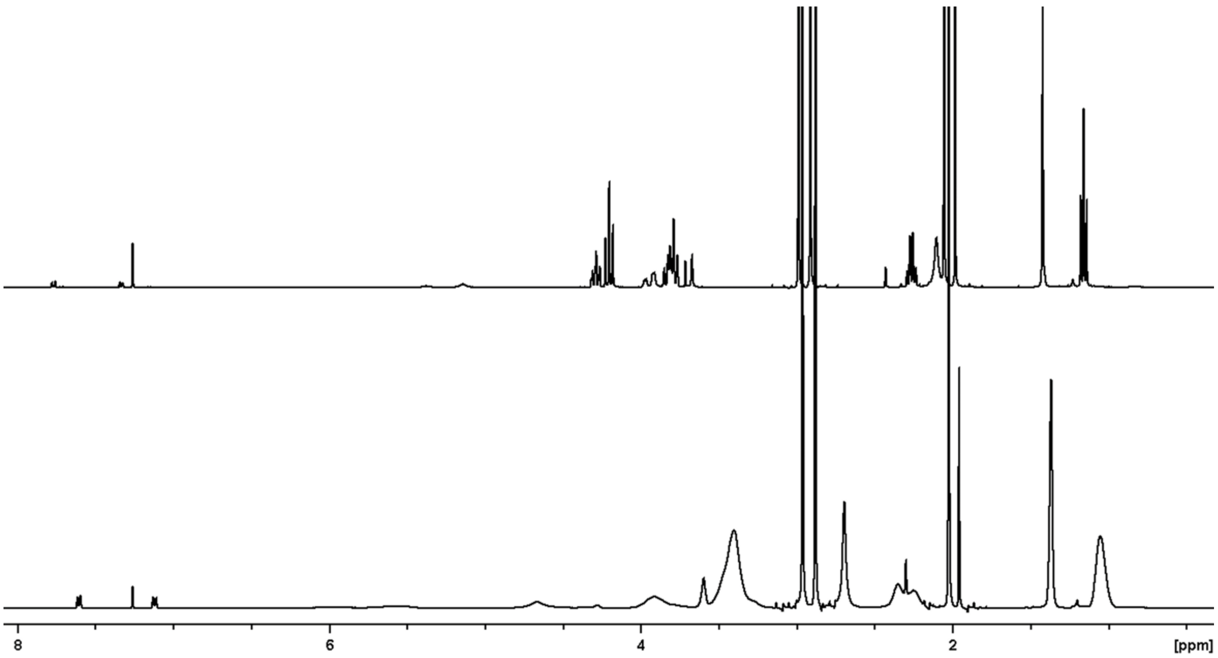

Figure S7. ^{1}H NMR spectra (400 MHz, $CDCl_3$) of C2-30 at t_0 (top) and t_f (bottom).

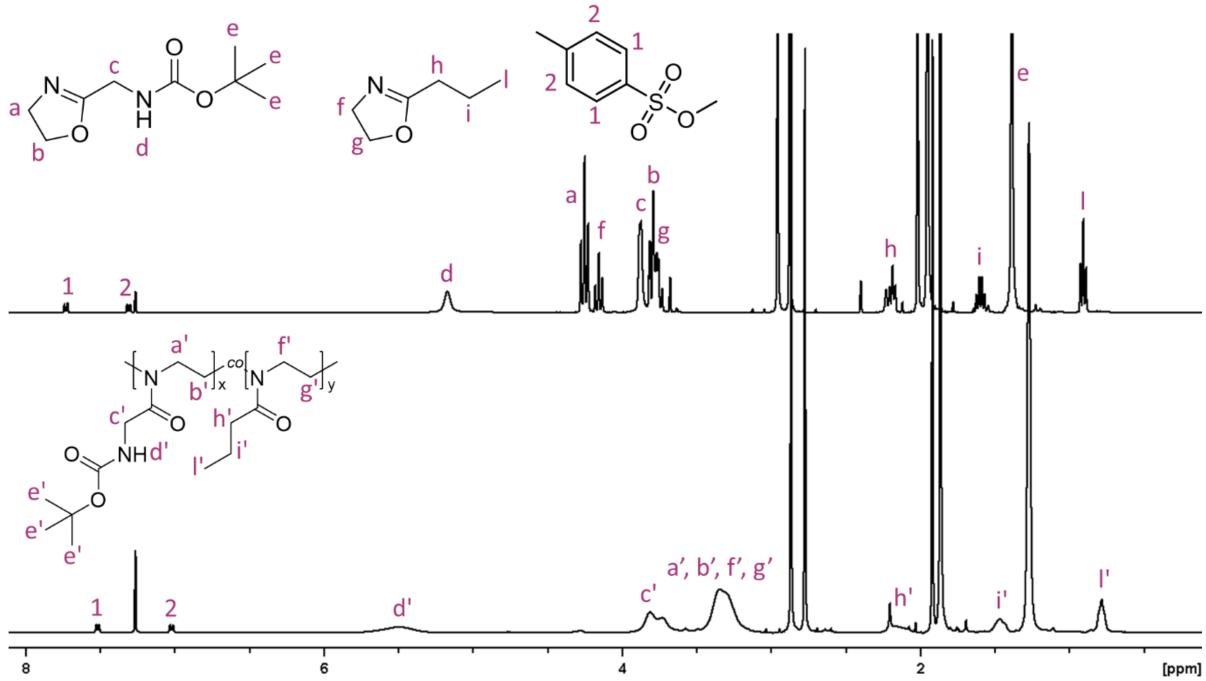

Figure S8. ^{1}H NMR spectra (400 MHz, $CDCl_3$) of C3-70 at t_0 (top) and t_f (bottom).

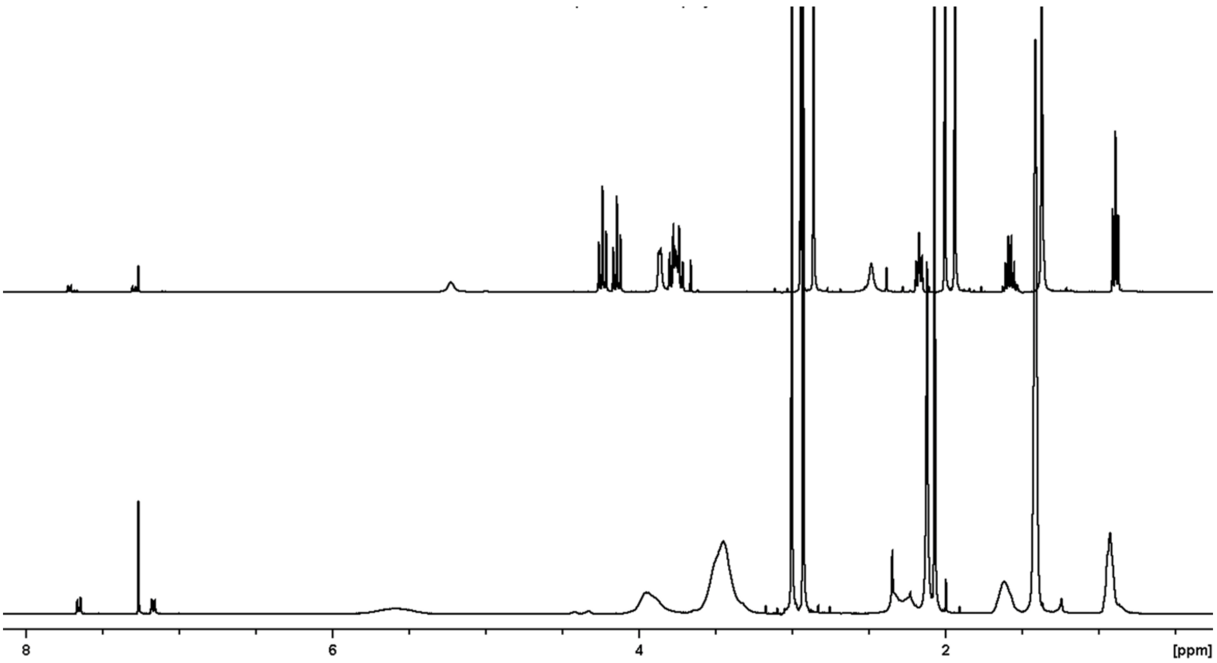

Figure S9. ^{1}H NMR spectra (400 MHz, $CDCl_3$) of C3-50 at t_0 (top) and t_f (bottom).

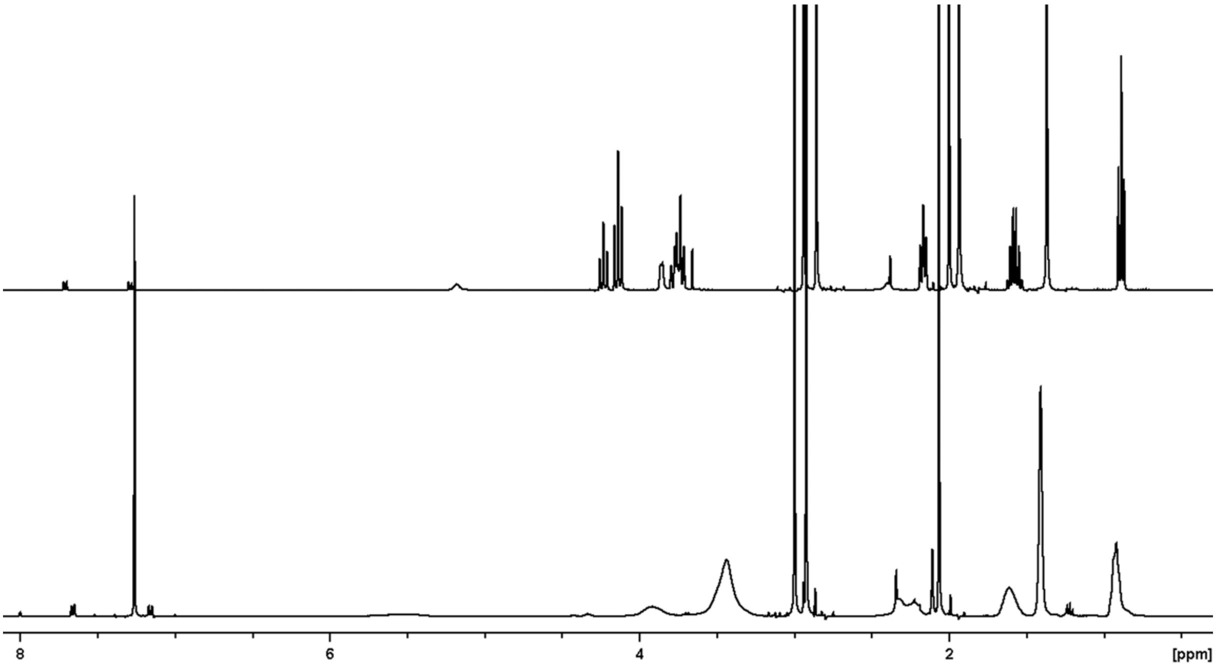

Figure S10. ^{1}H NMR spectra (400 MHz, $CDCl_3$) of C3-30 at t_0 (top) and t_f (bottom).

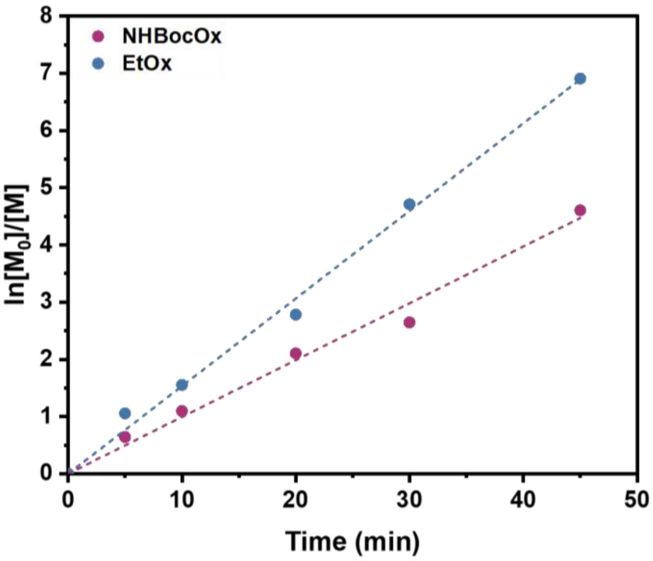

Figure S11. First order kinetic plot for the copolymerisation of NHBocOx and EtOx (CH_3CN:DMAc = 2:1, 100 °C, [M]= 2.13, MeTos as initiator, [M]/[I]= 16).

Table S2. Apparent propagation rate constants ($k_{p,app}$), propagation rate constants (k_p), and apparent reactivity ratios (r_1 and r_2) obtained for the copolymerisation of NHBocOx with EtOx.

Monomer 1	Monomer 2	$k_{pNHBocOx,app}$ (10^{-3} s^{-1})	$k_{pEtOx,app}$ (10^{-3} s^{-1})	$k_{pNHBocOx}$ (L mol^{-1} s^{-1})	k_{pEtOx} (L mol^{-1} s^{-1})	r_1 ($k_{pNHBocOx}/k_{pEtOx}$)	r_2 ($k_{pEtOx}/k_{pNHBocOx}$)
NHBocOx	EtOx	99.4±3.2	153.2±3.2	0.75±0.02	1.15±0.02	0.65	1.53

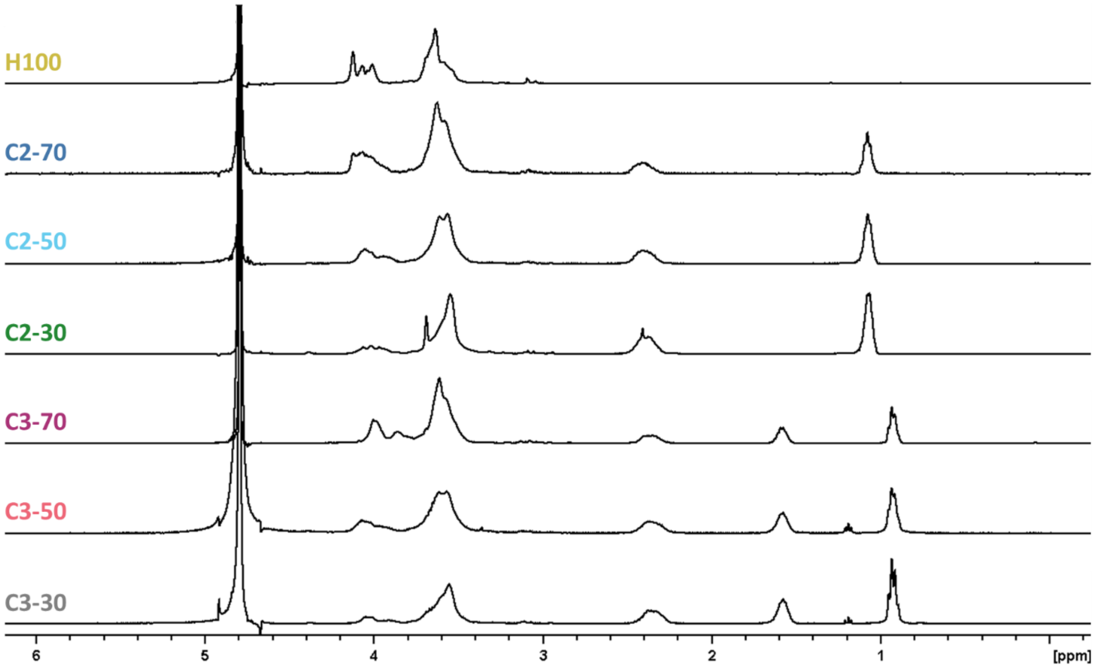

Figure S12. ^1H NMR spectra (400 MHz, D_2O) of the deprotected homopolymer and copolymers showing the complete removal of the Boc groups.

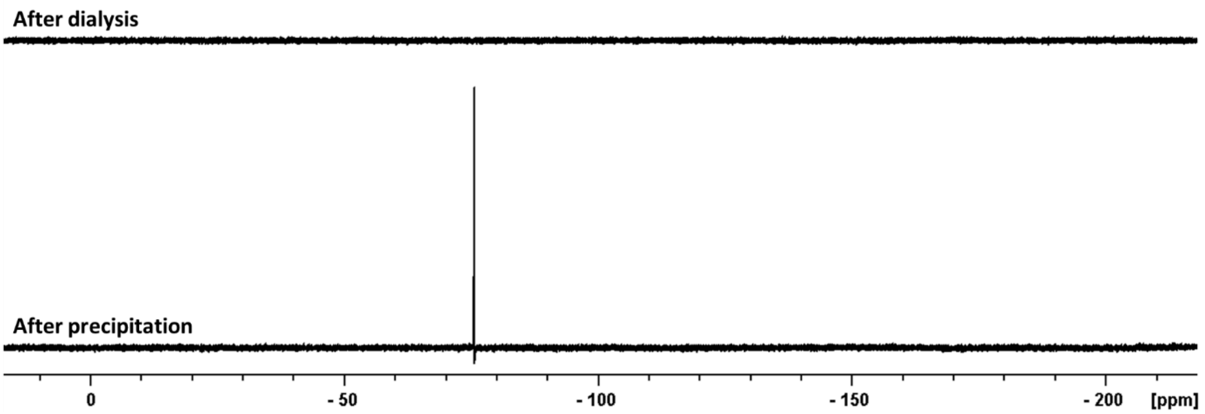

Figure S13. Example of ^{19}F NMR spectra (400 MHz, D_2O) of the deprotected C2-50 copolymer after precipitation (bottom) and after dialysis (top) showing the complete removal of the TFA counterion.

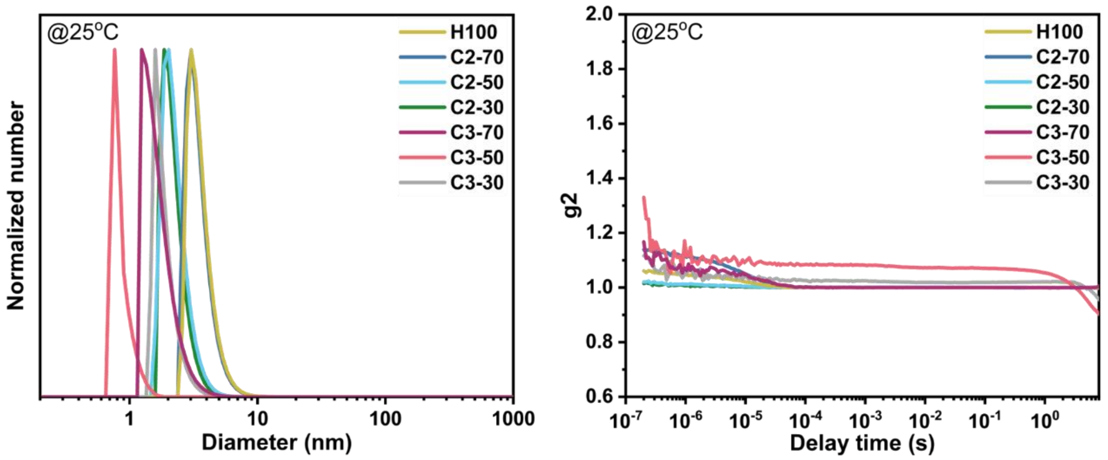

Figure S14. Normalized number DLS measurements and correlation curves of 1 mg mL^{-1} solutions in PBS of the deprotected homo and copolymers at 25 °C.

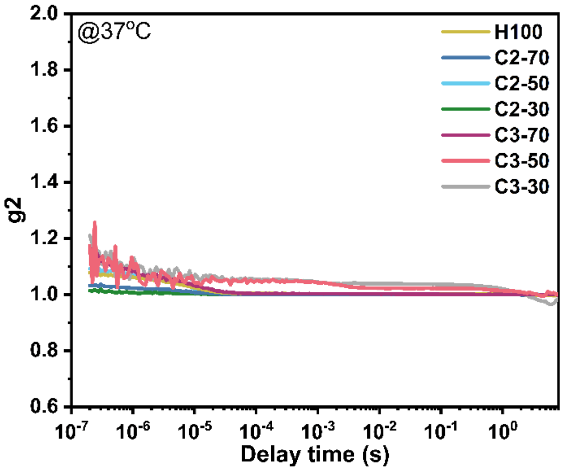

Figure S15. Correlation curves of 1 mg mL^{-1} solutions in PBS of the deprotected homo and copolymers at 37 °C.

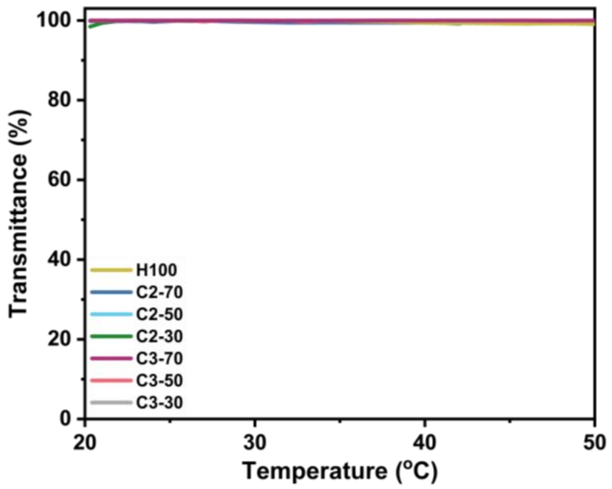

Figure S16. Turbidity curves of the second cooling cycle of 1 mg mL^{-1} solutions in PBS of the deprotected homo and copolymers measured at λ = 600 nm.

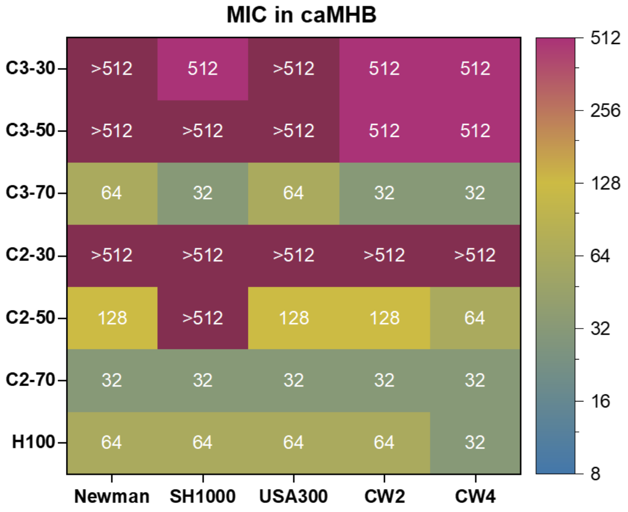

Figure S17. Heatmap of the MIC values (μg mL^{-1}) of the homo and copolymers tested against 5 different strains of *S. aureus* in caMHB. The colour gradient was used to highlight the most active compounds (blue) to the inactive ones (magenta).

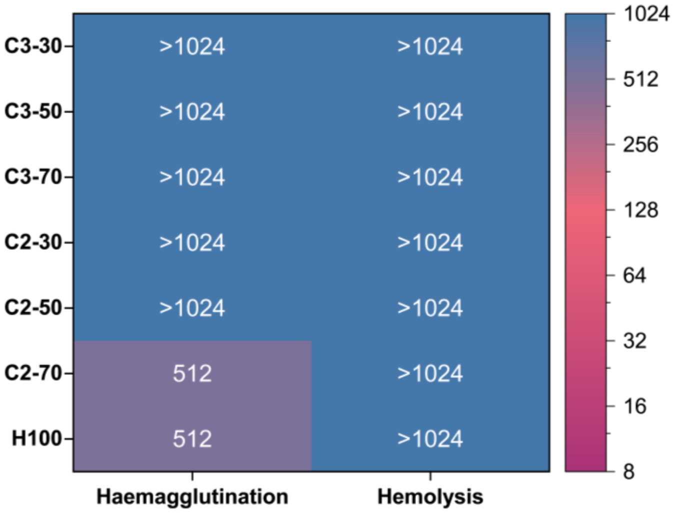

Figure S18. Heatmap of the haemagglutination and hemolysis of sheep RBCs in the presence of the homo and copolymers tested in PBS. The colour gradient was used to highlight the most toxic compounds (magenta) to the least toxic ones (blue).

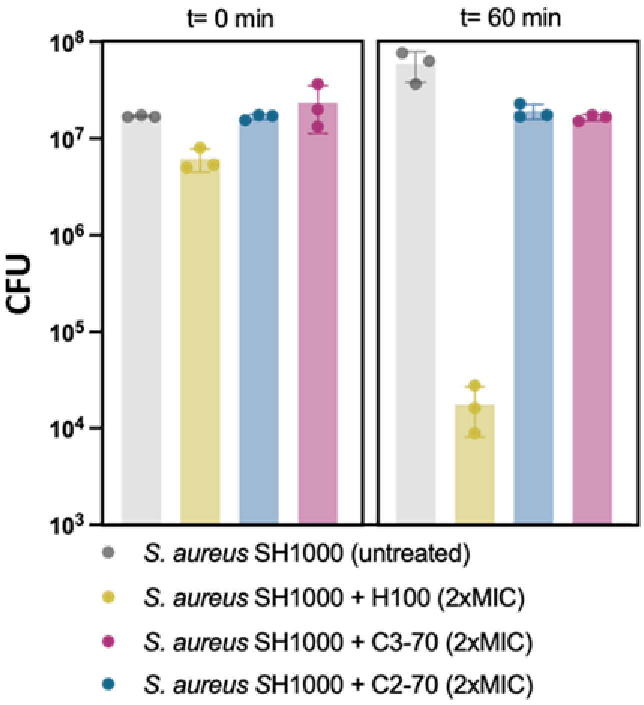

Figure S19. Colony count forming units from OD_{600} = 0.5 of *S. aureus* SH100 in caMHB at t = 0 and after 1 h exposure to H100 (yellow), C2-70 (blue), and C3-70 (magenta) at 2 x MIC concentration. Untreated *S. aureus* SH100 bacteria were used as control (grey). 3 biological independent experiments ± standard deviation are shown.

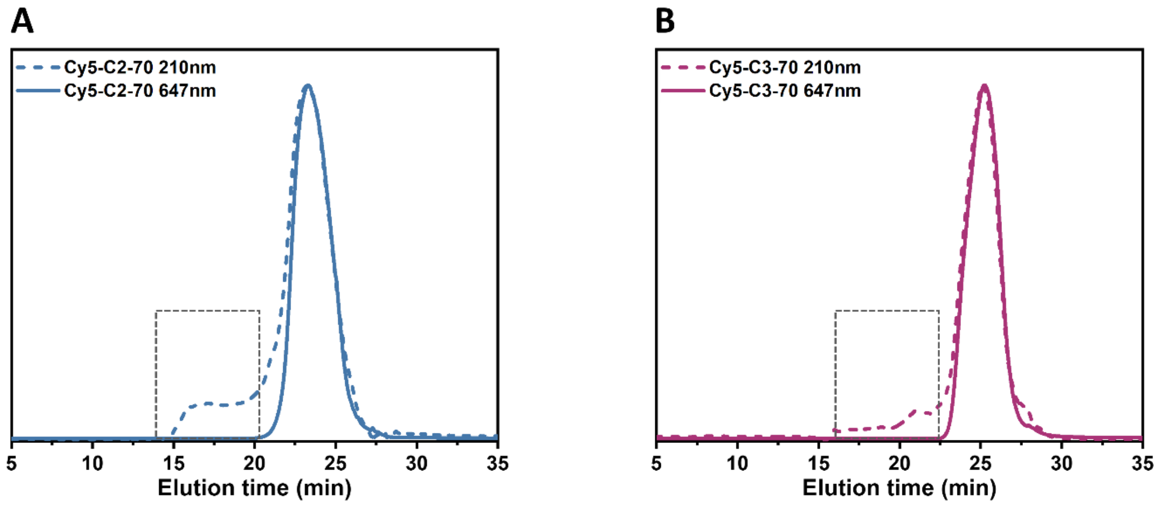

Figure S20. HPLC chromatograms of (A) Cy5-C2-70, and (B) Cy5-C3-70 measured at 210 nm (dashed line) and at 647 nm (solid line). The dotted rectangles highlight the non-conjugated polymer.

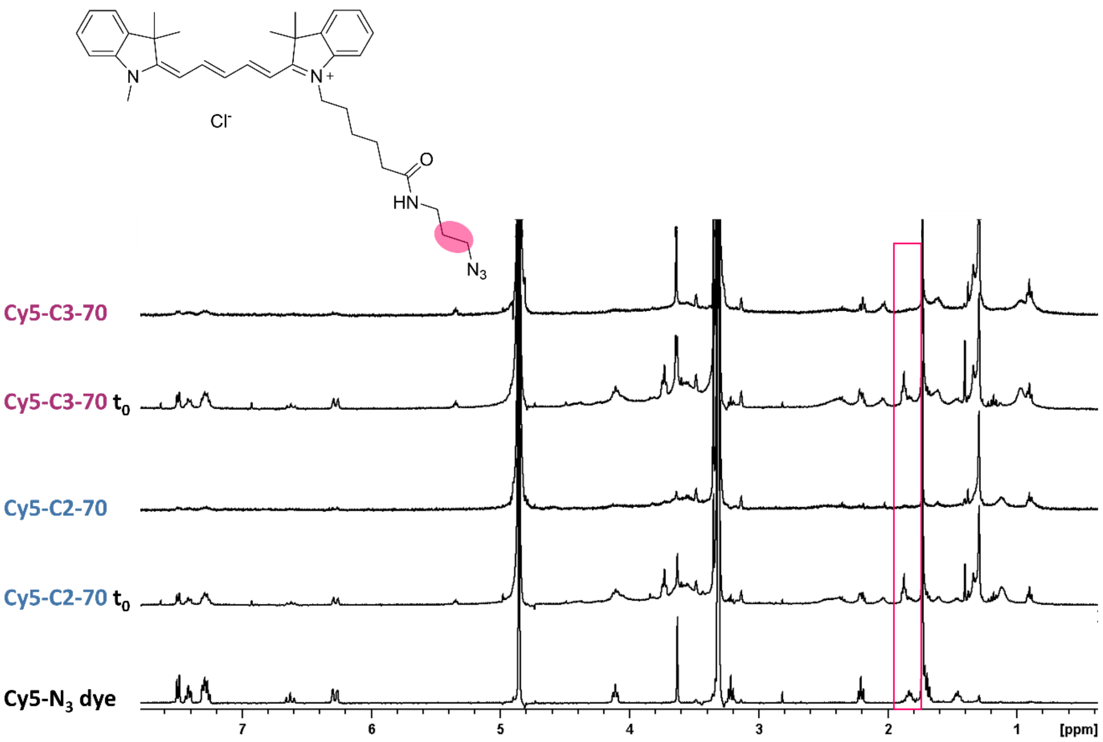

Figure S21. ^{1}H NMR spectra (500 MHz, MeOD) of the Cy5-N_3 dye and of the Cy5-polymers at t_0 and after the azide-alkyne "click" reaction, showing the disappearance of the peaks related to the free dye.

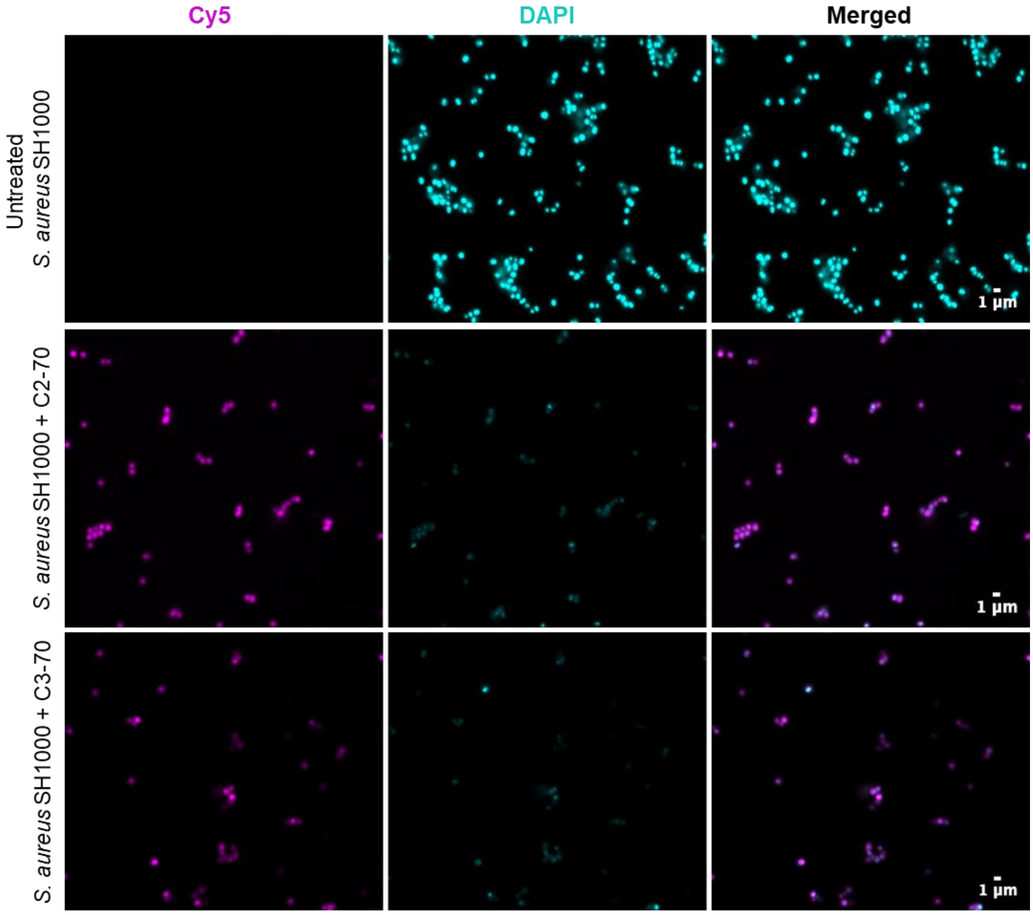

Figure S22. Representative confocal micrographs of *S. aureus* SH1000 untreated and treated with Cy5-C2-70 and Cy5-C3-70 at 2 x MIC concentration. The nucleic acid was stained with DAPI.

REFERENCES

1 Boerman, M. A., Van der Laan, H. L., Bender, J. C. M. E., Hoogenboom, R., Jansen, J. A., Leeuwenburgh, S. C., Van Hest, J. C. M. Synthesis of pH-and thermoresponsive poly (2-n-propyl-2-oxazoline) based copolymers. *J. Polym. Sci. A Polym. Chem.* **54**, 1573-1582 (2016).

2 Patel, J. B., Cockerill, F. R., Bradford A. P., Eliopoulos, G. M., Hindler, J. A., Jenkins, S. G., Lewis, J. S., Limbago, B., Miller, L. A., Nicolau, D. P., Powell, M., Swenson, J. M., Traczewski, M. M., Turnidge, J. D., Weinstein, M. P., Zimmer, B. L. M07-A10: Methods for Dilution Antimicrobial Susceptibility Tests for Bacteria That Grow Aerobically; Approved Standard—Tenth Edition. *CLSI (Clinical and Laboratory Standards Institute)* **35** (2015).

3 Elshikh, M., Ahmed, S., Funston, S., Dunlop, P., McGaw, M., Marchant, R., Banat, I. M. Resazurin-based 96-well plate microdilution method for the determination of minimum inhibitory concentration of biosurfactants. *Biotechnol. Lett.* **38**, 1015-1019 (2016).

4 Werthen, M., Henriksson, L., Jensen, P. Ø., Sternberg, C., Givskov, M., Bjarnsholt, T. An in vitro model of bacterial infections in wounds and other soft tissues. *Apmis* **118**, 156-164 (2010).

5 Banerjee, N., Sengupta, S., Roy, A., Ghosh, P., Das, K., Das, S. Functional alteration of a dimeric insecticidal lectin to a monomeric antifungal protein correlated to its oligomeric status. *PloS one* **6**, e18593 (2011).

6 Nečas, D., Klapetek, P. Gwyddion: an open-source software for SPM data analysis. *Open Phys.* **10**, 181-188 (2012).

7 Garcia Maset, R., Hapeshi, A., Hall, S., Dalgliesh, R. M., Harrison, F., Perrier, S. . Evaluation of the Antimicrobial Activity in Host-Mimicking Media and In Vivo Toxicity of Antimicrobial Polymers as Functional Mimics of AMPs. *ACS Appl. Mater. Interfaces* **XXXX**, XXX-XXX (2022).

8 Sheehan, G., Dixon, A., Kavanagh, K. Utilization of Galleria mellonella larvae to characterize the development of Staphylococcus aureus infection. *Microbiology* **165**, 863-875 (2019).
